# Effects of High-Intensity Interval Training on the Parameters Related to Physical Fitness and Health of Older Adults: A Systematic Review and Meta-Analysis

**DOI:** 10.1186/s40798-024-00767-9

**Published:** 2024-09-12

**Authors:** Wei Liang, Xiang Wang, Shishi Cheng, Jiao Jiao, Xiangui Zhu, Yanping Duan

**Affiliations:** 1 School of Physical Education, Shenzhen University, Shenzhen, China; 2 Department of Sport, Physical Education and Health, Hong Kong Baptist University, Hong Kong, China; 3 School of Physical Education, Hebei Normal University, Shijiazhuang, China

**Keywords:** HIIT, Physical fitness, Body composition, Cardiorespiratory fitness, Mobility, Elderly population

## Abstract

**Background:**

As a novel and time-efficient exercise form, high-intensity interval training (HIIT) has shown great potential in improving health-related physical fitness among diverse populations. However, empirical evidence on its efficacy among the elderly has not been well summarized. This systematic review and meta-analysis aimed to determine the effect of HIIT interventions on the parameters related to physical fitness and health of older adults, including resting heart rate (HR), systolic blood pressure (SBP), diastolic blood pressure (DBP), cardiorespiratory fitness (CRF), body mass index (BMI), body fat percent (BF%), waist circumference (WC), muscular endurance (ME), muscular strength (MS), muscular power (MP), balance and flexibility, compared to non-exercise and other-exercise (e.g., moderate-intensity continuous training, resistance training) conditions.

**Methods:**

Literature published from January 2000 to May 2023 was collected through extensive searches across eight databases and relevant review papers. Randomized controlled trials (RCTs) featuring a minimum 2-week exercise intervention for older adults (≥ 60 years) were included. The pooled effect size of Hedges’*g* was estimated using random-effects models in *R*. Meta-regression was performed for both categorical (health status, duration of training programme, and frequency) and continuous moderators (mean age, male rate, and attrition rate).

**Results:**

Forty-four eligible RCTs with 1863 participants (52.1% female; 60.5–81.2 years) were included in the quantitative analysis. Compared to non-exercise condition, HIIT significantly improved resting HR (*g* = -0.36, 95%CI = [-0.67, -0.05], *P* = 0.032), SBP (*g* = -0.29, 95%CI = [-0.54, -0.03], *P* = 0.008), CRF (*g* = 0.77, 95%CI = [0.51, 1.04], *P* < 0.001), BF% (*g* = -0.26, 95%CI = [-0.41, -0.11], *P* = 0.006), MS (*g* = 0.47, 95%CI = [0.23, 0.71], *P* = 0.004), ME (*g* = 0.65, 95%CI = [0.10, 1.19], *P* = 0.036), and balance (e.g., timed-up-and-go) (*g* = -0.79, 95%CI = [-1.19, -0.40], *P* = 0.035). Compared to other-exercise condition, HIIT significantly improved resting HR (*g* = -0.11, 95%CI = [-0.21, -0.01], *P* = 0.029), SBP (*g* = -0.14, 95%CI = [-0.28, -0.01], *P* = 0.038), and CRF (*g* = 0.23, 95%CI = [0.07, 0.38], *P* = 0.008). No significant difference was found between HIIT and non-exercise condition for DBP, BMI and WC, as well as between HIIT and other-exercise condition for DBP, BMI, BF%, WC, ME, and balance (all *P* > 0.05). Meta-regression indicated that mean age moderated the HIIT effect on resting HR (*b* = -0.02, *P* = 0.014; HIIT vs. other-exercise condition) and SBP (*b* = 0.03, *P* = 0.048; HIIT vs. non-exercise), and attrition rate moderated the effect on CRF (*b* = 0.03, *P* = 0.007; HIIT vs. non-exercise).

**Conclusion:**

This study supports the efficacy of HIIT in improving resting HR, SBP, CRF, BF%, MS, ME and balance among older adults. More empirical evidence is needed to determine the efficacy of HIIT for MP and flexibility in this population.

**Trial Registration:**

PROSPERO CRD42022316246.

**Supplementary Information:**

The online version contains supplementary material available at 10.1186/s40798-024-00767-9.

## Key Points

• HIIT is an effective approach for improving older adults’ resting heart rate (HR), systolic blood pressure (SBP), cardiorespiratory fitness (CRF), body fat percent (BF%), muscular strength (MS), and balance, compared with non-exercise condition.

• HIIT outperformed the other exercise interventions in improving older adults’ resting HR, SBP, CRF, and muscular endurance (ME).

• Mean age and attrition rate were identified as potential moderators for the HIIT effects on resting HR, SBP and CRF.

**Supplementary Information:**

The online version contains supplementary material available at 10.1186/s40798-024-00767-9.

## Background

Ageing is one of the greatest public health challenges faced by countries worldwide. The World Health Organization (WHO) has stated that the number and proportion of older adults aged ≥ 60 years is increasing rapidly. The number was 1 billion in 2019, and it is expected to increase to 1.4 billion by 2030 and 2.1 billion by 2050 [1]. This unprecedented increase in the ageing population is unavoidable and will accelerate in the coming decades, especially in developing countries [1]. It is known that a decline in physical fitness is a common health problem that accompanies ageing, which affects physical function, the risk of chronic diseases, and quality of life [2, 3]. According to the American College of Sport Medicine (ACSM), health-related physical fitness is defined as ‘a set of attributes that people have or achieve that relates to the ability to perform physical activity’, which is closely related to individuals’ physical, mental and social health [4]. The health-related components of physical fitness consist of cardiorespiratory fitness (CRF), body composition (e.g., body mass index [BMI], body fat percent [BF%], waist circumference [WC]), muscular endurance (ME), muscular strength (MS), muscular power (MP), balance and flexibility [4]. Traditionally, muscular power and balance should be considered as skill-related physical fitness components instead of health-related physical fitness components. However, the 11th ACSM’s guidelines suggest including muscular power in the assessment of muscular fitness. This is because muscular power tends to decline at a faster rate compared to muscular strength or muscular endurance with aging [5], and it may be the most significant of the muscular fitness variables for predicting maintenance of functional independence and improving quality of life [6]. Regarding balance, the ACSM position statement recommends that balance training is an effective way for fall prevention [7], which is closely related to aging health. Balance is increasingly becoming an additional component of health-related fitness [4]. Additionally, the ACSM’s guidelines emphasize that a comprehensive physical fitness assessment includes the measurement of resting heart rate (HR) and resting blood pressure (BP) [4]. Those two parameters closely relate to the health of older people as well. Therefore, the parameters related to physical fitness and health of older adults include resting HR, BP, CRF, BMI, BF%, WC, ME, MS, MP balance and flexibility in this study.

An overwhelming group of evidence has demonstrated that exercise training is a crucial part of healthy ageing and is conducive to improving health-related physical fitness of older adults [8, 9]. Moderate-intensity continuous training (MICT), as a ‘traditional’ aerobic exercise protocol, has been a leading exercise recommendation in older adults for nearly three decades [10, 11]. It refers to moderate intensity of effort (55–69% HR_max_ or 40–59% VO_2peak_) performed continuously at a steady state for a set duration [12]. MICT has been shown to be associated with a wide range of physical fitness and health indicators, including CRF, BP and body composition [13–15]. While traditional exercise programmes can offer numerous benefits, their implementation can be challenging for older adults, mainly because of their long duration, which often leads to diminished engagement, motivation, and compliance with the exercise prescription [16]. In this scenario, high-intensity interval training (HIIT), which is suggested as an alternative to traditional MICT, has attracted increasing interest in recent years. As a novel and time-efficient exercise form, HIIT consists of repeated bouts of high-intensity exercise that last seconds to minutes, interspersed with periods of rest [17, 18]. Similar to traditional MICT, HIIT can include diverse forms of exercise modalities such as cycling, dancing, treadmill running, jumping-based exercise etc. [19]. The main distinction is that HIIT involves alternating short bursts of vigorous exercise (lasting from 10 s to 5 min) that typically elevate one’s heart rate to at least 80% of their maximum capacity (HR_max_), with recovery periods of rest or light exercise (lasting no more than 5 min) at ≤ 70% HR_max_ [19, 20]. A typical HIIT session lasts about half the duration of a MICT session [11, 15]. The feasibility, safety, and tolerability of HIIT programmes amongst older adults have been demonstrated by a recent scoping review [19]. In addition, previous studies have found that HIIT can improve older adults’ CRF [21, 22], body composition [23], muscle fitness [24], metabolic parameters [14], cognitive function [25, 26], and mental health [27].

Recently, several reviews have provided preliminary evidence on the effect of HIIT in improving older adults’ physical fitness [11, 28, 29]. However, most of them focused mainly on the CRF of older adults [28, 29], while evidence on the other crucial parameters of health-related physical fitness and health (e.g., resting HR, WC, MP, balance, and flexibility) has not been well summarized. Furthermore, existing reviews have shown several methodological limitations. For example, a recent meta-analysis demonstrated that HIIT has a significant medium effect on functional movement, as assessed using the 30-s chair sit-to-stand test (STS) and 8-foot (timed) up and go (TUG) test in older adults compared with the non-intervention group [30]. This review used standardized mean difference (SMD) to pool effect sizes, which may have introduced upward bias because of the inclusion of several studies with sample sizes lower than 20 [31]. Another review measured seven outcomes (i.e., 6-min walking test [6MWT], TUG test, chair test, upper limb MS, lower limb MS, MP, and citrate synthase activity) to examine the effect of HIIT [28]. Although this review showed that HIIT had significant effects on the 6MWT, TUG test, chair test, MP, and citrate synthase activities, the consistency of the results might have been influenced by two substantial shortcomings. First, four of the experimental groups included in the review used a HIIT intervention combined with other approaches, such as resistance training and a nutritional strategy. Second, some of the included studies did not satisfy the randomized controlled trials (RCTs) criteria because of the lack of a control group. Additionally, existing reviews reported conflicting findings regarding the effects of HIIT on the resting BP of older adults. Carpes et al. found that HIIT significantly decreased the systolic BP (SBP) and diastolic BP (DBP) of older adults compared with the non-intervention group, whereas another meta-analysis found no such significant effect [32]. Overall, the conflicting outcomes and variations in research methods across these studies indicate the need for further studies on this topic.

Therefore, the purpose of this systematic review and meta-analysis was to identify the evidence on and quantify the impact of HIIT interventions on a wide range of parameters related to physical fitness and health (i.e., resting HR, resting BP, body composition, CRF, ME, MS, MP, balance, and flexibility) in both clinical and non-clinical older adults compared with other-exercise (e.g., MICT, resistance training) and non-exercise control conditions. In addition, the following specific characteristics of interest were tested as moderators of the effects of HIIT: participants’ characteristics (e.g., age, male-to-female ratio, health status, and attrition rate) and intervention characteristics (e.g., frequency and duration of the training sessions).

## Methods

### Protocol and Registration

This study was conducted following the Reporting Items for Systematic Reviews and Meta-Analyses (PRISMA) 2020 statement [33]. The protocol was registered in the International Prospective Register of Systematic Reviews (PROSPERO) database (Prospero ID: CRD42022316246). The PROSPERO database and Cochrane Library of systematic reviews were searched for existing or pending systematic reviews and meta-analyses to avoid duplication.

### Identification of Studies and Search Strategy

A systematic literature search of eight electronic databases (i.e., Medline, PsycINFO, SPORTDiscus, Scopus, Embase, CT.gov, the Cochrane Library, and PubMed) was conducted. Article titles and abstracts were searched using the key terms that were generated from a summary of previous review papers and commonly used synonyms for HIIT (see Supplementary file 1). To enable a more specific search, the following limitations were applied: (1) English language, (2) human subjects, (3) journal articles, and (4) published from 1st January 2000 to 31st May 2023 (given prior reviews indicating that HIIT has primarily been utilized in health promotion fields since the beginning of the 21st century [19, 30, 32], this systematic review commenced the literature search from the year 2000). In addition to the structured database search, literature from the bibliographies of relevant review articles was searched by hand. Two authors (XW & SC) completed the systematic search for articles and the removal of duplicates. The titles and abstracts of the remaining articles were then screened by the same two authors.

### Inclusion and Exclusion Criteria

The full texts of the articles were screened for inclusion by two authors (XW & SC). Another author (WL) was consulted in cases of doubt or disagreement between the first two authors. The full-text screening was conducted based on the following PICOS criteria.

1. *Participants*: The mean age of participants was ≥ 60 years, and there were no restrictions regarding participants’ demographics and medical conditions.
2. *Interventions*: The intervention protocol included at least one group performing HIIT intervention, defined as brief, intermittent bursts of vigorous activity interspersed with periods of low-to-moderate-intensity activity or rest [17]. Exercise intensity is commonly assessed based on oxygen uptake (VO_2_), HR, and heart rate reserve (HRR). High intensity was categorised as ‘very hard’ effort (≥ 90% of peak HR; ≥ 85% of HRR; ≥ 80% of peak VO_2_) or ‘vigorous’ effort (70%-89% of peak HR; 60%-84% of HRR; 60%-79% of peak VO_2_) [19, 34]. To increase the generalisability of the findings, we also included the studies that used perceived exertion of at least 16 on the Borg scale to define high intensity. Additionally, the minimum duration of the intervention was set at 2 weeks to allow for the capture of training adaptations rather than just the acute effects [30]. No limitations were set for other intervention characteristics (e.g., exercise mode, frequency, and recovery mode).
3. *Comparators*: The comparator groups included in the studies were mainly another exercise intervention group (e.g., the MICT intervention) or a non-exercise control group.
4. *Outcomes*: The studies evaluated at least one of the following outcomes: resting HR, resting BP (usually SBP and DBP), CRF (usually relative VO_2max_ or VO_2peak_), body composition (usually BMI, BF%, and WC), MS (usually the chair stand test and grip strength test), ME (usually the sit-to-stand test and arm curl test), MP (usually the countermovement vertical jump test), balance (usually the TUG test), and flexibility (usually the chair sit and reach test, and back scratch test).
5. *Study design and article type*: Individual or cluster RCTs were included. Reviews, editorial and commentaries, non-peer-reviewed papers, and non-English papers were excluded.

### Risk of Bias and Certainty of Evidence

Two authors (CS & WL) independently evaluated the risk of bias for the included studies using version 2 of the Cochrane Risk of Bias Tool (RoB2) [35], based on five criteria: (1) randomisation process, (2) deviations from the intended interventions, (3) missing outcome data, (4) measurement of the outcome, and (5) selection of the reported results. For each criterion, the risk of bias was judged as low, some concerns, or high. Based on the assessment, the quality of the included studies was classified into three levels: low risk, some concern and high risk.

Additionally, the quality and certainty of the evidence was determined using the Grading of Recommendations Assessment, Development and Evaluation (GRADE) system [36]. Specifically, the quality was downgraded in case of each of the following limitations: imprecision of results (wide 95% confidence intervals [CIs]), inconsistency of results (*I*^2^ values > 50% were considered to indicate substantial heterogeneity) [37], indirectness of evidence (indirect population, intervention, control, and outcomes), risk of bias (> 50% studies with more than one item with a high risk of bias) [37], and high probability of publication bias (Egger’s regression test results being significant). After two authors independently evaluated the quality of evidence according to the criteria, consensus was reached on the ratings and the overall quality of the summary statistics. Any disagreement between the two authors was resolved by discussion or consultation with a third investigator (XW/YD).

### Data Extraction

Data extraction from the included articles was conducted by two authors (XW & SC). The extracted data included basic information about the study, study methodology, participants’ characteristics, intervention characteristics, and measurement outcomes. The specific variables were authors, publication year, participants’ health condition (i.e., healthy older or older adult patients), the country and common setting (refers to the environment or context in which the HIIT exercise was conducted), study design, sampling method, sample size, age (mean and standard deviation [SD]), sex (male rate), attrition rate, attendance rate, exercise type, recovery mode (i.e., active or passive), intensity assessment (i.e., objective or subjective), duration of the training programme, frequency, volume of HIIT, durations of HIIT intervals (i.e., work interval and rest interval), interval repetitions, warm-up time, cool-down time, and statistical data for outcome variables (i.e., *n*, mean, and SD). The intervention groups were categorized as the HIIT group, other-exercise group (engaged in an exercise training programme other than HIIT), and control group (not engaged in any training programme). In cases of articles missing relevant data, their authors were contacted via email for the missing data.

### Summary of Measures

The primary outcomes assessed in this review were resting HR, resting BP, CRF, body composition (i.e., BMI, BF % and WC), MS, ME, MP, balance, and flexibility. The moderators were participants’ characteristics (age, male rate, health status, and attrition rate), and intervention characteristics (duration of the training programme, frequency of exercise sessions, recovery mode and exercise mode). These descriptive data were also summarized in this review (Table 1).

**Table 1:** Characteristics of studies and subjects included in the review

Study	Groups	Sample size	M/F	Country	Age	BMI	Other population characteristics
					(years)	(kg/m2)	
Aboarrage et al. [64]	HIIT	15	0/15	Brazil	65 ± 7	30 ± 5	Postmenopausal women
	CON	10	0/10			27 ± 7	
Adamson et al. [80]	SIT_1	11	4/7	UK	66 ± 4	27.1 ± 4.2	
	SIT_2	11	5/6		65 ± 4	26.8 ± 4.1	
	CON	12	2/10		65 ± 3	26.0 ± 4.3	
Adamson et al. [85]	SIT	10	6/4	UK	66 ± 4	26.9 ± 3.5	Inactive and without any metabolic disease or cardiovascular disease
	CON	7	3/4		66 ± 2	25.9 ± 3.3	
Angadi et al. [53]	HIIT	9	8/1	USA	69.0 ± 6.1	29.8 ± 5.1	HFpEF diagnosis 85 with New York Heart Association heart failure Class II-III symptoms
	MICT	6	4/2		71.5 ± 11.7	29.3 ± 2.8	
Ballesta-García et al. [76]	HIIT	17	0/17	Spain	66.3 ± 5.4	30.4 ± 4.1	
	MICT	12	0/12		70 ± 8.8	30.1 ± 3.1	
	CON	12	0/12		67.4 ± 5.7	31.2 ± 4.9	
Ballin et al. [75]	HIIT	36	35/37	Sweden	70.7 ± 0.2	29.2 ± 3.3	
	CON	36					
Boa Sorte Silva et al. [25]	HIIT	65	33/32	Canada	71.7 ± 6.3	29.3 ± 5.8	Subjective cognitive decline (SCD) as defined by scoring 26 on the Montreal Cognitive Assessment (MoCA)
	MICT	63	34/29		70.4 ± 7.1	30.3 ± 6.5	
Boukabous et al. [23]	HIIT	9	0/9	Canada	66.0 ± 3.4	30.1 ± 4.9	Abdominal obesity
	MICT	9	0/9		64.2 ± 3.7	31.7 ± 3.5	
Brown et al. [81]	HIIT	33	16/17	Australia	70.2 ± 5.3	25.8 ± 3.7	
	MICT	34	16/18		68.4 ± 4.2	26.0 ± 3.9	
	CON	32	13/19		68.7 ± 5.9	25.3 ± 3.4	
Bruseghini et al. [67]	HIIT	12	12/0	Italy	69.4 ± 4.3	26.5 ± 2.8	
	MICT	12	12/0		69.67 ± 4.1	26.8 ± 2.9	
Coetsee and Terblanche [71]	HIIT	13	3/10	South Africa	64.5 ± 6.3	26.6 ± 4.0	Inactive older adults
	MICT	13	3/10		61.6 ± 5.8	26.5 ± 4.2	
	RT	22	7/15		62.4 ± 5.1	25.8 ± 4.0	
	CON	19	8/11		62.5 ± 5.6	26.9 ± 3.7	
Coswig et al. [14]	HIIT	15	0/15	Brazil	80.3 ± 5.8	25.6 ± 2.2	All participants were residents of the same nursing home and were there for social reasons or familiar decision
	MIIT	15	0/15		80.9 ± 4.6	26.1 ± 1.7	
	MICT	16	0/16		81.2 ± 5.4	25.9 ± 1.9	
Currie et al. [22]	HIIT	11	NA	Canada	62 ± 11	27.9 ± 4.9	Patients with coronary heart disease CAD
	MICT	11	NA		68 ± 8	27.3 ± 4.2	
de Matos et al. [58]	HIIT	58	20/38	Brazil	64.5 ± 4.8	F: 26.9 ± 6.2	Metabolic syndrome and hyper triglyceridemic waist phenotype in older adults
						M: 26.9 ± 3.4	
	CON	46	21/25		65.6 ± 4.0	F: 26.8 ± 7.0	
						M: 27.7 ± 1.5	
Fu et al. [52]	HIIT	14	9/5	Taiwan	67.5 ± 1.8	NA	Heart failure patient
	MICT	13	8/5		66.3 ± 2.1	NA	
	CON	13	9/4		67.8 ± 2.5	NA	
Herrod et al. [77]	HIIT	13	5/8	UK	71 ± 4	NA	
	IHG	11	3/8			NA	
	CON	12	7/5			NA	
Hurst et al. [72]	HIIT	16	11/7	UK	61.9	28.1 ± 4.4	
	CON	16	10/8		62.8	27.4 ± 5.3	
Hwang et al. [68]	HIIT	18	5/9	USA	64.8 ± 1.4	28.0 ± 1.1	Sedentary, non-smokers
	MICT	18	7/7		65.6 ± 1.8	28.7 ± 1.0	
	CON	14	5/10		63.8 ± 1.6	26.7 ± 1.4	
Hwang et al. [59]	HIIT	14	9/9	USA	65 ± 2.0	31.7 ± 1.3	Diagnosis of type 2 diabetes
	MICT	15	10/6		62 ± 2.0	31.8 ± 1.4	
	CON	18	8/8		61 ± 2.0	33.9 ± 1.4	
Iellamo et al. [57]	HIIT	17	16/2	Italy	67.2 ± 6.0	28.3 ± 3.0	Patients with CHF secondary to coronary artery disease (CAD)
	MICT	16	15/3		68.4 ± 8.0	28.1 ± 2.0	
Izadi et al. [60]	HIIT	15	8/7	Iran	60.5 ± 6.3	25.2 ± 1.0	Older treated hypertensive individuals
	CON	15	9/6		62.4 ± 5.5	25.4 ± 0.6	
Jiménez-García et al. [73]	HIIT	26	2/24	Spain	68.2 ± 3.0	29.5 ± 3.7	
	MIIT	24	7/17		68.5 ± 6.0	30.3 ± 3.1	
	CON	23	8/15		68.5 ± 6.3	32.1 ± 2.3	
Kim et al. [74]	HIIT	14	NA	USA	65 ± 1	28.1 ± 1.2	
	MIIT	13	NA		65 ± 2	28.7 ± 1.0	
	CON	11	NA		63 ± 2	25.3 ± 1.4	
Klonizakis et al. [66]	HIIT	11	0/11	UK	64 ± 7.0	NA	Postmenopausal women
	MICT	7	0/7		64 ± 4.0	NA	
Kovacevic et al. [26]	HIIT	21	7/14	Canada	72.4 ± 4.4	27 ± 3	Sedentary
	MICT	20	10/10		72 ± 6.2	26 ± 3	
	CON	23	8/15		71.5 ± 6.6	26 ± 2	
Krawcyk et al. [61]	HIIT	31	23/7	Denmark	63.7 ± 8.9	28 ± 5	With first-time lacunar stroke or a recurrent event of lacunar stroke were enrolled in the study
	CON	32	26/6		63.7 ± 9.2	26 ± 4	
Li et al. [69]	HIIT	10	7/3	Chinese	64.9 ± 3.5	27.8 ± 1.0	Physically inactive overweight or obese volunteers
	MICT	10	6/4		66.4 ± 4.5	27.7 ± 2.8	
	CON	9	5/4		63.9 ± 4.0	27.1 ± 1.5	
Maillard et al. [86]	HIIT	8	0/8	France	61 to 80	32.6 ± 1.7	Overweight women with T2DM
	MICT	8	0/8			29.7 ± 1.2	
Mekari et al. [62]	HIIT	24	9/15	Canada	67 ± 5	26 ± 4	
	MICT	19	9/10		68 ± 7	26 ± 4	
	RT	26	9/17		67 ± 7	26 ± 7	
Nilsson et al. [24]	HIIT	38	31/9	Norway	68.8 ± 7.9	NA	Patients with chronic heart failure
	CON	38	32/8		71.5 ± 7.8	NA	
Northey et al. [82]	HIIT	6	0/6	Australia	60.3 ± 8.1	NA	Breast cancer survivors, female
	MICT	5	0/5		67.8 ± 7.0	NA	
	CON	6	0/6		61.5 ± 7.8	NA	
Nunes et al. [65]	HIIT	13	0/13	Brazil	62.3	31.4	Obese postmenopausal women
	CT	13	0/13		62.9	30.6	
O’Brien et al. [63]	HIIT	12	5/7	Canada	68 ± 5	25.9 ± 3.1	
	MICT	12	4/8		68 ± 6	25.2 ± 3.6	
	RT	14	6/8		66 ± 7	27.2 ± 5.1	
Reed et al. [55]	HIIT	43	29/14	Canada	68 ± 8	30.9 ± 5.7	Patiens with atrial fibrillation
	CON	43	26/17		71 ± 7	29.9 ± 6.2	
Rognmo et al. [56]	HIIT	8	6/2	Norway	62.9 ± 11.2	26.7 ± 4.1	Patients with coronary artery disease
	MICT	9	8/1		61.2 ± 7.3	26.9 ± 2.7	
Santos et al. [87]	HIIT	9	0/9	Brazil	69.1 ± 5.0	NA	Pre-frail older women
	MICT	11	0/11		69.7 ± 5.6	NA	
Sculthorpe et al. [107]	HIIT	22	22/0	UK	62.3 ± 4.1	NA	Sedentary male volunteers
	CON	11	11/0		61.6 ± 5.0	NA	
Sian et al. [78]	L-HIIT	10	5/5	UK	70 ± 5.0	26 ± 3.0	
	H-HIIT	10	3/7		71 ± 4.0	25 ± 3.0	
	CON	10	6/4		71 ± 7.0	26 ± 1.0	
Simonsson et al. [79]	HIIT	34	15/19	Sweden	69.7 ± 3.2	26.1 ± 3.5	
	MIIT	33	15/19		69.6 ± 2.8	26.4 ± 4.4	
Spee et al. [54]	HIIT	12	12/0	Netherlands	68.9 ± 6.7	NA	Participants with HF selected for cardiac resynchronization therapy
	CON	12	7/5		68.8 ± 6.5	NA	
Terada et al. [83]	HIIT	8	4/4	Canada	62 ± 3	28.4 ± 4.1	Type 2 diabetes
	MICT	7	4/3		63 ± 5	33.1 ± 4.5	
Varga et al. [84]	HIIT	17	11/6	USA	67 ± 10	25 ± 4	
	HICT	22	19/3		61 ± 12	26 ± 4	
	MICT	32	25/7		60 ± 12	25 ± 4	
Wisløff et al. [51]	HIIT	9	7/2	Norway	76.5 ± 9	24.5 ± 3	Stable postinfarction heart failure
	MICT	9	7/2		74.4 ± 12	24.7 ± 3	
	CON	9	6/3		75.5 ± 13	25.5 ± 2	
Wyckelsma et al. [70]	HIIT	8	6/2	Australia	69.4 ± 3.5	21.6 ± 2.6	
	CON	7	3/4				

*Note*: HIIT = high-intensity interval training, L-HIIT = Laboratory-high-intensity interval training, H-HIIT = Home-high-intensity interval training, HICT = High-intensity continuous training, MICT = Moderate-intensity continuous training, MIIT = Moderate-intensity interval training, CT = Combined training, CON = No exercise intervention control group

### Meta-Analysis

For the effect size calculation and data analysis procedure, Cohen’s*d* (also known as the SMD) [38] values were calculated for all 229 sets of data identified in 44 studies. For studies that reported both pre-test and post-test outcomes of the experimental and control groups, change scores were used to calculate the effect sizes. The following formulas were used:

1 $${d}_{1}=(\bar{\text{x}}1-\bar{\text{x}}2)/{\text{S}}_{\text{p}\text{o}\text{o}\text{l}\text{e}\text{d}}$$

2 $${\text{S}}_{\text{p}\text{o}\text{o}\text{l}\text{e}\text{d}}=\sqrt{\frac{(n1-1){s1}^{2}+(n2-1){s2}^{2}}{\left(n1-1\right)+(n2-1)}}$$

3 $$SE=\sqrt{\frac{n1+n2}{n1n2}+\frac{{d}^{2}}{2(n1+n2)}}$$

4 $${S}_{E,Change}=\sqrt{\begin{array}{l}{{\text{S}\text{D}}_{E,\text{b}\text{a}\text{s}\text{e}\text{l}\text{i}\text{n}\text{e}}^{2}+{\text{S}\text{D}}_{E,\text{f}\text{i}\text{n}\text{a}\text{l}}^{2}}\\{-(2\times Corr\times{\text{S}\text{D}}_{E,\text{b}\text{a}\text{s}\text{s}\text{e}\text{l}\text{i}\text{n}\text{e}}\times{\text{S}\text{D}}_{E,\text{f}\text{i}\text{n}\text{a}\text{l}})}\end{array}}$$

where $\stackrel{-}{\text{x}}1$, and $\stackrel{-}{\text{x}}2$ were obtained by subtracting the post-test mean from the pre-test mean; $n1$ and $n2$ are the sample sizes of the two groups, respectively; the SD of change (i.e., $s1$ and $s2$) was transformed using the following the formula given below; and *Corr* (correlation coefficient) was assumed to be 0.5 between the baseline and follow-up measurements [35]. Notably, Cohen’s *d* for effect size has been found to have an upward bias when the study sample size is small, especially when *n* ≤ 20 [31]. Therefore, all the Cohen’s values were converted into Hedges’ *g* by using the following formula suggested by Hedges (1981) to correct for overestimation [31].

5 $$\text{G}=d\times(1-\frac{3}{4n-9})$$

We examined whether there were outliers to provide evidence of the robustness of the findings. For each effect size, values that were outside the interval of $\stackrel{-}{\text{x}}$ – 2sd and $\stackrel{-}{\text{x}}$ +2sd were considered outliers [39], and analyses were repeated without these effects. Meta-analyses were conducted if at least three datasets provided effect sizes of HIIT for the same outcome [40]. A random-effects model was used to pool the overall effect size of HIIT because we assumed that the true effect size could vary from study to study [41]. The Tau^2^ and *I*^2^ statistic was used to assess the heterogeneity across studies [42, 43]. Tau^2^ is the estimate of between-study variance of the group. A larger Tau^2^ value indicates higher heterogeneity beyond what would be expected by chance alone [43]. *I*^2^ values of < 25%, 50%, and 75% indicated low, moderate, and high heterogeneity, respectively [42]. For pooling effect sizes that were significant (*P* < 0.05) or had higher values of heterogeneity and at least 8 datasets [44], meta-regression was used to examine the moderator for explaining the variability.

Finally, Egger’s regression test was used to identify the existence of publication bias [44]. If publication bias was found (i.e., *P* < 0.1 in Egger’s regression test), the selection model by Vevea and Woodds was used to obtain an overall effect size corrected by publication bias [45]. In this method, the adjusted overall effect size was measured by specifying a weight function of the probability of being published assigned to the observed effect size according to their *P* values. The weights for the weight function were selected based on those suggested by Vevea and Woods for moderate one-tailed selection [45]. In addition, sensitivity analysis was performed using the leave-one-out function, in which the meta-analysis is performed by removing one effect size at a time. These removed effect sizes were from studies with high risks of bias.

All of the analyses were performed in *R* (version 4.3.0, *R* Foundation for Statistical Computing). Specifically, the effect sizes were calculated using *dmetar* [46] and *tidyverse* packages [47], the main meta-analysis was conducted using the *meta* package [48], the meta-regression was performed using the *metafor* package [49], and publication bias was examined using the *weightr* package [50].

## Results

### Study Selection

**Fig. 1:**
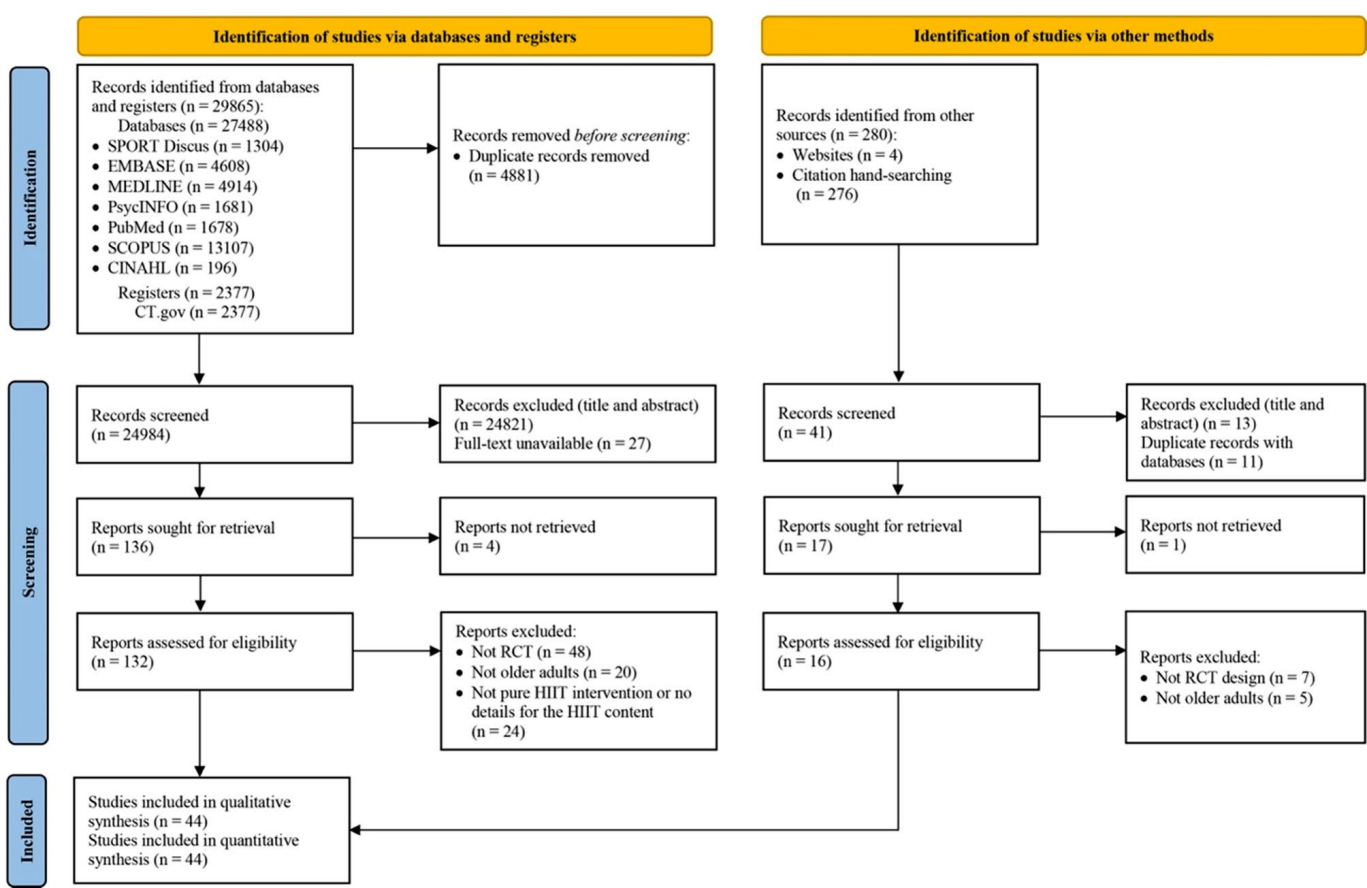
PRISMA flow diagram

Literature searches were performed in eight databases, which resulted in a total of 29,865 articles. Following the removal of 4881 duplicates, the titles and abstracts of the remaining 24,984 articles were screened. Of these, 132 full-text articles were screened for eligibility, and 92 were excluded for several reasons as shown in Fig. 1. Additionally, 280 articles were collected from other sources (website and reference lists of relevant review articles), of which 16 were included in the eligibility check. Finally, a total of 44 studies satisfied the inclusion criteria for both qualitative and quantitative analyses (Fig. 1).

### Study Characteristics

Participants’ characteristics from included studies are outlined in Table 1. A total of 1863 participants (872 male and 970 female; two studies did not provide sex information) from 15 countries were divided into HIIT groups (*n* = 821), other-exercise groups (i.e., MICT, moderate-intensity interval training [MIIT], resistance training [RT], combined training [CT], high-intensity continuous training [HICT]; *n* = 513) or non-exercise control groups (*n* = 529). The attrition rates ranged from 0 to 35.29% in the HIIT groups (the rate was < 20% in 37 studies), 0–35.29% in the other-exercise groups (the rate was < 20% in 26 studies), and 0–34.29% in the non-exercise control groups (the rate was < 20% in 25 studies). In addition, 20 studies reported the attendance rates of the HIIT groups (the rate was > 80% in 17 studies), and 14 studies reported the attendance rates of the other-exercise groups (the rate was > 80% in nine studies). The mean age ranged from 60.6 to 81.2 years, and the mean BMI ranged from 21.6 to 33.9 kg/m^2^. The studies included patients with heart failure [24, 51–54], atrial fibrillation [55], subjective cognitive decline [25], coronary artery disease [22, 56, 57], metabolic syndrome and hyper-triglyceridemic waist phenotype [58], type 2 diabetes [59], hypertension [60], and lacunar stroke [61].

HIIT protocol features from included studies are outlined in Table 2. In terms of the intervention design, the training lasted between 2 weeks to 24 weeks, with 12 weeks being the most common duration (*n* = 18), and training sessions were conducted one to five times per week. The HIIT training included work intervals ranging from 6 s to 4 min, rest intervals ranging from 12 s to 4 min, cool-down and warm-up times ranging from 2 to 15 min, and exercise modes consisting of treadmill (*n* = 12), cycling (*n* = 21), and others (e.g., sprints, resistance exercises, and comprehensive exercise) (*n* = 11). Regarding work-to-rest ratio (WRR) of HIIT, the most widely applied ratio was 1:1 (*n* = 15) including 15s:15s [62, 63], 30s:30s [55, 64], 1 min:1 min [22, 58, 65, 66], 2 min:2 min [67], 3 min:3 min [52, 59, 68, 69], 4 min:4 min [14, 70], followed by 4:3 (*n* = 11) including 4 min:3 min [25, 26, 51, 53, 54, 56, 57, 71–74]. HIIT implementation settings included the laboratory, fitness centers, healthcare facilities, university and home. The laboratory setting was the most common (*n* = 25), followed by the healthcare facilities setting (*n* = 5) and the fitness centre setting (*n* = 4).

The outcome measures were: resting HR assessed with the 12-lead electrocardiograph (ECG) (*n* = 6) and blood pressure measurement (*n* = 1), photoelectric pulse wave method (*n* = 4), resting blood pressure (SBP and DBP), cardiorespiratory fitness (VO_2peak_, VO_2max_) assessed with a treadmill test (*n* = 9), cycle ergometer test (*n* = 16) and Chester step test (*n* = 1), BMI, WC, BF% assessed with the dual-energy X-ray absorptiometry method (*n* = 10), bioelectrical impedance analysis (*n* = 2) and skinfold caliper (*n* = 1), MS assessed with the chair stand test (*n* = 5), handgrip strength test (*n* = 3) and maximal isometric knee extensor muscle strength (*n* = 1), ME assessed with the 6MWT (*n* = 5), loaded 50 m walk test (*n* = 1), Bruce protocol (*n* = 1), MP assessed with the Nottingham leg extensor power rig (*n* = 1) and step test (*n* = 1), balance assessed with the TUG (*n* = 6), static balance (*n* = 1) and standing on one leg with eyes closed (*n* = 1), flexibility assessed with the sit and reach test (*n* = 1).

Twenty-five studies reported no adverse events during HIIT, and six studies reported adverse events (one study did not provide detailed information [73]) including: (1) Achilles tendinitis, lateral epicondylitis, knee bursitis, muscle strain, and swelling in the metacarpophalangeal joint [75]; (2) low-back pain, hip soreness, hypertensive crisis, knee soreness, and muscle soreness [25]; (3) shortness of breath, dizziness and hypotension [59] (4) nausea/vomiting, knee swelling/medial collateral ligament tear, and uncontrolled HR [55]; (5) an abnormally high blood pressure response to exercise and mild vasovagal episodes [70]. Thirteen studies did not provide relevant information on adverse events.

### Risk of Bias in the Included Studies

**Fig. 2:**
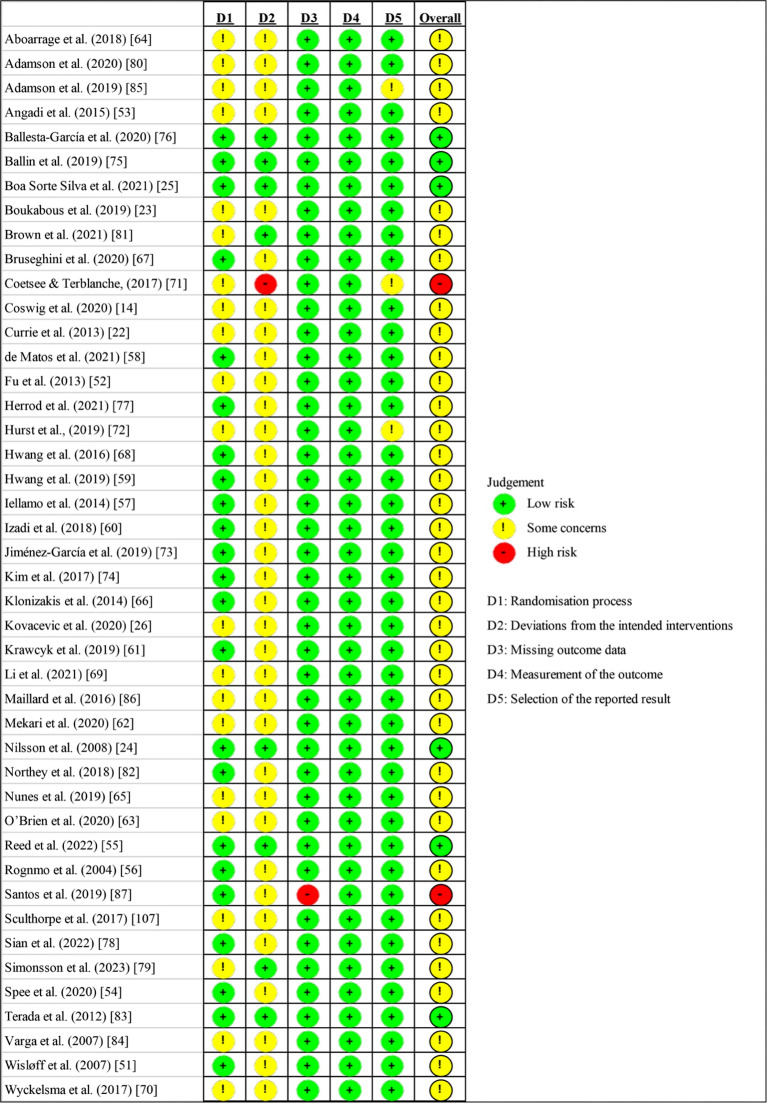
Risk of bias assessment for all included studies

As outlined in Fig. 2, six studies were judged to be at a low risk of bias, and 36 studies were assessed as having some concerns. The main possible bias was the lack of pre-registration of intentions/methodology, which was particularly relevant for the overall results. In addition, two studies were considered to have a high risk of bias due to the possible bias in deviations from the intended interventions and missing outcome data (12 out of 34 participants discontinued the intervention).

### Resting Heart Rate

*HIIT Versus Non-exercise* The effect of HIIT versus non-exercise groups on the resting HR outcome was investigated in six studies (*n* = 217) [52, 59, 60, 74–76]. Table 3 shows a small significant effect (*g* = -0.358, 95%CI = [-0.671, -0.045]) of the HIIT interventions with a moderate-GRADE quality of evidence (Table 4). No heterogeneity was evident.

*HIIT Versus Other-exercise* The effect of HIIT versus other-exercise groups on resting HR was investigated in 13 studies (*n* = 478) [14, 22, 25, 51, 52, 55–57, 59, 63, 66, 74, 76]. The HIIT groups showed a significant reduction in resting HR (*g* = -0.113, 95%CI = [-0.213, -0.014]) (Table 1) with a high-GRADE quality of evidence (Table 3). No statistically significant heterogeneity was identified.

### Resting Blood Pressure

*HIIT Versus Non-exercise* The effect of HIIT versus non-exercise conditions on the SBP outcome was investigated in 10 studies (*n* = 340) [52, 59–61, 70, 74–78], in which the HIIT groups showed a significant reduction in SBP (*g* = -0.287, 95%CI = [-0.542, -0.032]) (Table 3). No statistically significant heterogeneity was observed across the studies, but the result was of a low GRADE quality of evidence (Table 4). Furthermore, the effect of HIIT versus non-exercise groups on the DBP outcome was investigated in 8 studies (*n* = 291) [52, 60, 61, 70, 74–76, 78]. The HIIT groups showed a small non-significant reduction in DBP (*g* = -0.249, 95%CI [-0.606, 0.108]) (Table 3). A small heterogeneity was evident (*Tau*^*2*^ = 0.09, *I* = 36.1%). The result was of a moderate GRADE quality of evidence (Table 4).

*HIIT Versus Other-exercise* The effect of HIIT versus other-exercise groups on the SBP outcome was investigated in 17 studies (*n* = 693) [14, 22, 23, 25, 52, 53, 55–59, 63, 66, 74, 76, 77, 79]. The HIIT groups also showed a significant reduction in SBP compared with the other-exercise groups (*g* = -0.143, 95%CI = [-0.277, -0.009]) (Table 3). No heterogeneity was evident. In addition, the effect of HIIT versus active group on the DBP outcome was investigated in 15 studies (*n* = 621) [14, 23, 25, 52, 53, 55–59, 63, 66, 74, 76, 79]. The HIIT groups showed a trivial non-significant reduction in DBP relative to the other-exercise groups (*g* = -0.022, 95%CI = [-0.172, 0.128]) (Table 1). No statistically significant heterogeneity was noted across the studies. These results provide high and moderate GRADE quality of evidence for the SBP and DBP outcomes, respectively (Table 4).

### Cardiorespiratory Fitness

*HIIT Versus Non-exercise* The effect of HIIT versus non-exercise control on the CRF outcome was investigated in 14 studies (*n* = 413) [26, 54, 59, 68–70, 72, 74, 76, 78, 80–82], in which the HIIT groups showed a large, significant increase in CRF relative to the control groups (*g* = 0.774, 95%CI = [-0.506, 1.041]) (Table 1) with a moderate-GRADE quality of evidence (Table 3). Only trivial heterogeneity was observed among these studies (*Tau*^*2*^ = 0.07, *I*^2^ = 26.6%).

*HIIT Versus Other-exercise* The effect of HIIT versus other-exercise groups on the CRF outcome was investigated in 19 studies (*n* = 572) [22, 23, 26, 52, 53, 56, 57, 59, 63, 66–69, 74, 79, 81–84]. The HIIT groups also showed a small but significant increase in CRF relative to the other-exercise conditions (*g* = 0.228, 95%CI = [0.067, 0.383]) (Table 3) with a high-GRADE quality of evidence (Table 4). No heterogeneity was evident.

### Body Composition

*HIIT Versus Non-exercise* The between-group differences in the effects of HIIT versus non-exercise control group on BMI, BF%, and WC are shown in Table 3. Eleven studies (*n* = 354) [59, 61, 64, 68, 69, 73, 74, 76, 78, 80, 85] evaluated BMI, seven studies (*n* = 241) [59, 64, 68, 69, 73, 78, 81] evaluated BF%, and three studies [59, 73, 74](*n* = 108) evaluated WC. Overall, the pooled effect size illustrated a significant difference between the HIIT and non-exercise groups in terms of BF% (*g* = -0.257, 95%CI = [-0.406, -0.108]), but non-significant differences in BMI (*g* = -0.127, 95%CI = [-0.266, 0.014]) and WC (*g* = -0.155, 95%CI = [-0.462, 0.152]). The GRADE quality of pooled effect size for BF% was high, and that for the other outcomes was moderate (Table 4).

*HIIT Versus Other-exercise* The between-group difference in the effects of HIIT and other-exercise interventions on BMI, BF%, and WC were evaluated in 15 studies (*n* = 479) [23, 53, 55, 59, 62, 63, 65, 67–69, 73, 74, 76, 83, 86], 11 studies (*n* = 410) [14, 55, 59, 65, 67–69, 73, 81, 83, 86], and eight studies (*n* = 271) [23, 55, 59, 67, 73, 74, 83, 86], respectively. According to Table 3, the pooled effect size illustrated a non-significant difference between the HIIT and other-exercise interventions in terms of BMI (*g* = -0.086, 95%CI = [-0.202, 0.031]), BF% (*g* = -0.064, 95%CI = [-0.186, 0.059]), and WC (*g* = 0.027, 95%CI = [-0.066, 0.120]). No further analyses were performed because the values of *Tau*^*2*^ and *I*^2^ for all these pooled effects were 0.

### Muscle Fitness and Mobility

*HIIT Versus Non-exercise* The between-group differences in the effects of HIIT versus non-exercise control groups on ME, MS, and balance are shown in Table 3. Four studies (*n* = 115) [64, 69, 72, 80] evaluated MS, two studies tested (*n* = 81) [64, 80] ME, and five studies (*n* = 125) [64, 69, 71, 80, 85] evaluated balance. Overall, the pooled effect size demonstrated significant differences between the HIIT and non-exercise condition in terms of MS (*g* = 0.469, 95%CI = [0.225, 0.713]), ME (*g* = 0.648, 95%CI = [0.101, 1.194]) and balance (*g* = -0.794, 95%CI = [-1.187, -0.401]). No heterogeneity was evident for the outcomes of MS, ME and balance.

*HIIT Versus Other-exercise* The between-group differences in the effects of HIIT versus other-exercise interventions on MS, ME, and balance were evaluated in five studies (*n* = 165) [14, 23, 69, 79, 87], four studies [14, 23, 55, 71] (*n* = 159), and four studies (*n* = 104) [23, 69, 71, 87], respectively. According to Table 3, the pooled effect size demonstrated a significant difference between the HIIT and other-exercise interventions in terms of MS (*g* = 0.272, 95%CI = [0.028, 0.516]), but non-significant differences in ME (*g* = 0.158, 95%CI = [-0.074, 0.389]) and balance (*g* = 0.008, 95%CI = [-0.724, 0.741]). No heterogeneity was evident for the outcomes of MS and ME, and a low heterogeneity was evident for balance (*Tau*^*2*^ = 0.12, *I*^2^ = 41.8%). The GRADE quality of pooled effect size for MS was low, and that for the other outcomes was moderate (Table 4).

### Meta-Regression Outcome

The meta-regression conducted on the significant pooled results of the meta-analysis revealed that mean age significantly moderated the effects on resting HR (HIIT vs. other-exercise condition, *n* = 16, *b* = -0.021, *P* = 0.014) and SBP (HIIT vs. non-exercise control, *n* = 11, *b* = 0.034, *P* = 0.042), and that attrition rate significantly moderated the effects on CRF (HIIT vs. non-exercise control, *n* = 14, *b* = 0.027, *P* = 0.012). The other moderators, including the male rate, health status, duration of the intervention programme, and frequency of exercise sessions, showed no significant moderating effects on any outcome measures (Supplementary file 2, Table S1).

### Publication Bias and Sensitivity Analysis

Egger’s regression test was used to identify potential publication bias. A significant publication bias is indicated if the intercept corresponds to *P* < 0.10; otherwise, there is no publication bias. As outlined in Table 2, publication bias was identified for SBP in the HIIT versus non-exercise comparison (*P* = 0.06) and for MS in the HIIT versus other-exercise comparison (*P* = 0.06). The selection model of Vevea and Woods [45] indicated that the adjusted pooled effects of HIIT on SBP and MS were − 0.323 and 0.204, respectively. Therefore, the overall effects observed on SBP may be somewhat deflated, and those on MS may be somewhat inflated. Furthermore, sensitivity analyses were conducted on the results that showed heterogeneity. According to the results (see Supplementary file 3), the heterogeneity of the pooled effect size for DBP (HIIT vs. non-exercise control) decreased to 0% after one study [60] was removed, but the significance or direction of the overall effect was not changed. The sensitivity analyses showed the same pattern of results for CRF and ME (HIIT vs. non-exercise control) after two studies [71] were removed respectively. However, in the results for balance (HIIT vs. other-exercise condition), the *g* value for heterogeneity decreased to 0% and the direction of the overall results changed (but remained nonsignificant) after one study [87] was excluded.

## Discussion

### Summary of the Characteristics of Included Studies

To the best of our knowledge, this is the first study to conduct a comprehensive systematic review and meta-analysis of the evidence on the effects of HIIT interventions versus non-exercise and other-exercise conditions on the parameters of physical fitness and health of older adults. Compared with the literature on the benefits of traditional exercise for older adults, the research on HIIT interventions is limited. The current review found that there was an increasing trend of HIIT interventions for older adults over the past decade, with most of the relevant studies (32/44, 72.7%) having been conducted in the last 7 years. The current analyses included 44 studies involving a total of 1863 participants (872 male, 970 female) aged 60 years and above from 15 countries, with BMIs ranging from 21.6 to 33.9 kg/m^2^. Our findings showed that compared with both non-exercise control and other-exercise interventions, HIIT was an effective approach for improving parameters related to physical fitness and health, including resting HR, SBP, CRF, BF%, MS, ME, and balance among older adults. Notably, our study observed considerable variability in the characteristics of HIIT interventions (e.g., frequency, duration, and content of intervention). This diversity likely reflects the varied objectives and health requirements of the studies included in our review. Such variability emphasizes the adaptability of HIIT protocols, enabling practitioners to tailor these interventions to meet the specific needs and preferences of individuals, thereby enhancing their practical application and effectiveness.

Despite the high variability in the included studies, some trends are notable. First, a majority of the HIIT intervention studies were conducted in Western countries (41/44, 93.2%), with only three studies conducted in Asia, i.e. China (*n* = 2) [52, 69] and Iran (*n* = 1) [60]. A possible explanation for this might be that HIIT originated in the West [88] and has a relatively well-developed training model [17]. Therefore, the HIIT intervention is mostly adopted in Western countries. Another possible explanation is that several non-English reviews or dissertations were not included in this review because they did not meet the inclusion criteria.

Second, most studies (36/44, 81.8%) have used objective methods to monitor the intensity of the HIIT intervention, such as ECG belts and wrist photoplethysmogram (PPG) sensors. Objective methods are more commonly used during HIIT because the intensity of HIIT is typically high and can be difficult to judge subjectively. HIIT requires individuals to push themselves to their limits during the high-intensity intervals, and thus, subjective results vary in accuracy based on the individual’s perception of exertion [89]. In addition, some participants may report dishonest data to show they are performing well. In contrast, objective monitoring methods, such as HR monitoring, provide accurate and measurable data on the individual’s physiological responses to the exercise intensity [90]. These data can help the individual to adjust their exercise intensity to ensure that they are exercising at the appropriate intensity to derive maximum benefit and avoid overexertion or injury.

Third, the most commonly adopted methods to facilitate HIIT were cycle ergometers and treadmills (32/44, 72.7%), which is in line with a previous study [19]. These types of exercises (e.g., using these devices in the laboratory) are easy to perform for older adults who desire exercise to be part of their daily lives. This is also consistent with an earlier review that showed that cycle ergometer training is particularly suitable for older adults because of its benefits for CRF, endurance parameters, and BP and because it is safer and exerted lower pressure on the joints than other typical components of exercise programmes [91]. However, it is worth noting that implementing these types of exercise in a real-world context that targets a larger sample size of populations can be challenging. Additionally, exercises performed using machines are limited in their ability to fully develop the essential skills of balance, gait and coordination that are vital to the daily lives of older adults [92]. Therefore, developing and evaluating a HIIT protocol tailored to meet the needs of older adults in a real-world setting, incorporating a group-based, machine-free exercise format, with supervised multifaceted exercise movements and greater enjoyment levels, is warranted in the future.

Although nearly 80% of included studies applied active recovery mode during HIIT, it was difficult to conclude that active recovery mode was superior to passive recovery mode. The latest systematic review investigated the effect of active or passive recovery mode in long-term interval training on physical fitness, and demonstrated that regardless of recovery mode, long-term interval exercise training has the potential to improve health-related physical fitness in adults [93]. A similar result was found in this study. There were no significant differences between active recovery and passive recovery of effect on health-related physical fitness outcomes (e.g., CF, BF %) in older adults (more details, see Supplementary file 2). Therefore, the participants and researchers can use either active or passive recovery mode when conducting HIIT programmes. The decision should be based on various factors, such as participants’ fitness level, and exercise workload.

Additionally, HIIT appears to be safe among older adults. Although only 70% (31/44) studies reported on adverse outcomes, this figure was higher than the previous review (58%, 7/12) [94], and 81% (25/31) studies explicitly reported that there was no adverse event during HIIT intervention. For six studies with adverse outcomes [25, 55, 59, 70, 73, 75], there were no serious adverse events requiring hospitalization or medical treatment. In addition, most adverse cases were resolved within the duration of study and none lasted throughout the entire intervention period [25, 59, 70, 73, 75]. For example, after resting and rehydrating with water, participants quickly recovered from shortness of breath and dizziness during HIIT and returned to the subsequent exercise without any problem [59]. Thirty percent of the included studies did not report any information on adverse events. This omission could introduce bias into the aggregated results, potentially skewing them towards a more favourable outcome than might be warranted [94]. Consequently, our findings cannot definitively ascertain the safety of HIIT for the elderly. Recognizing this gap, we advocate for future HIIT interventions to prioritize safety considerations. This can be achieved through meticulous protocol design involving healthcare professionals, rigorous scientific monitoring of participants, and the implementation of safety and emergency measures. Such strategies are vital to ensuring the well-being of participants and to bolster the credibility and acceptability of HIIT as a safe exercise modality for older adults.

### Effects on Physical Fitness and Health

As expected, this review found that HIIT significantly improved CRF in older adults, compared with other-exercise interventions (*g* = 0.23) and non-exercise controls (*g* = 0.76). This is in line with a meta-analysis by Wu et al. who reported that VO_2peak_ increased substantially after more than 12 weeks of HIIT at 2 sessions/week compared with MICT (weighted mean difference [WMD] = 1.74 ml/kg/min) and non-exercise controls (WMD = 2.28 ml/kg/min) [28]. Bouaziz et al. also found that HIIT led to a greater improvement in the VO_2peak_ of older adults aged ≥ 65 years compared with endurance training (mean difference [MD] = 3.76 ml/kg/min) and no-intervention control (MD = 4.61 ml/kg/min) [95]. This indicates that HIIT may be an effective way to increase aerobic capacity in older adults, as it induces greater central and peripheral adaptations than other traditional exercise modalities [95]. A possible explanation for this result might be improvement of the oxidative pathway due to increased muscle work during HIIT. Oxidative phosphorylation reduces lactate accumulation during interval periods. Training-induced reductions in blood lactate levels also lead to a slower breakdown of glycogen, which in turn favours more efficient oxidative pathways [17, 96, 97]. Notably, our meta-regression analysis showed that greater increases in VO_2peak_ were associated with a higher attrition rate. A possible explanation for this relationship between attrition rate and the effect of HIIT on VO_2peak_ is related duration of the HIIT programme. Based on common understanding in the field, the longer intervention periods yield better positive results [98]. However, the results of the meta-analysis did not support this. A possible explanation is the diversity of the HIIT protocol. Further empirical research is needed to identify the differential effects of intervention durations.

In addition, the current review found a significant reduction in resting HR after HIIT relative to other-exercise interventions (*g* = -0.11) and no-intervention controls (*g* = -0.36). To the best of our knowledge, this is the first systematic review and meta-analysis to assess the effect of HIIT on the resting HR of older adults. Our results are similar to those of a previous meta-analysis that identified the effect of HIIT on overweight/obese adults [99] and of school-based HIIT on children and adolescents [100]. The reduction in resting HR might be related to the increase in stroke volume and improvement of cardiac autonomic function via increased baroreflex-mediated modulation of the sinoatrial node [101, 102]. Our meta-regression analysis showed a greater reduction in resting HR in participants of older mean age. This result might be related to the baseline physical fitness level. Compared with the relatively low mean age group (mean age from 62 to 67, resting HR from 55 to 70 beats per minute (bpm); mean value of resting HR was 63.7 bpm) [22, 56, 59, 66, 74], the older mean age group had a higher resting HR at baseline (mean age from 67.2 to 80.8, resting HR from 55 to 76.9 bpm; mean value of resting HR was 69.6 bpm) [14, 25, 51, 57, 63]. To some extent, resting HR is a viable alternative measurement for monitoring physical fitness [103]. Therefore, the effect of HIIT on resting HR is more prominent in participants with poor physical fitness.

There is ongoing debate regarding the effectiveness of HIIT in reducing BP among older adults. A recent meta-analysis demonstrated that HIIT elicited a significant reduction in the BP of older adults when compared with the no-intervention group, whereas HIIT and MICT resulted in similar reductions in BP [32]. However, another study reported no correlation between HIIT and reductions in BP among older adults [28]. In this review, compared with the other-exercise intervention and no-intervention controls, HIIT showed a greater lowering effect on SBP. The characteristics of the included participants may be one of the reasons for these inconsistent results. In our study, the participants comprised both older adults with cardiovascular diseases or type diabetes and those without, whereas the HIIT group in the previous study that reported no significant effect included only healthy individuals. The possible mechanism of the BP-lowering effect of HIIT may involve an intensity-dependent increase in blood flow velocity, resulting in elevated levels of endothelial nitric oxide (NO) [104]. This increase in endothelial NO availability and bioactivity enhances NO-dependent vasodilation in the vasculature, leading to improved peripheral compliance and reduced BP [105]. Furthermore, our meta-regression analysis showed that the effect of HIIT on SBP declined with age. Age-related declines in physical fitness and exercise capacity may play a role in the observed decline in the effect of HIIT on SBP. As individuals age, they may experience declines in muscle mass, strength, and endurance, which could potentially reduce their ability to perform high-intensity exercise and experience the full benefits of HIIT [106, 107]. This explanation is somewhat speculative, as the exact mechanisms behind the effectiveness of HIIT in older populations are not fully understood. Overall, our study suggests that HIIT is a promising physical training intervention to improve SBP in older adults.

**Table 2:** HIIT protocol characteristics of the studies included in the systematic review

Study	Mode/Type	Common setting	Intensity	Work-out duration	Recovery time	Work rest ratio	Repetition	Duration (weeks)	Frequency (per week)	
Aboarrage et al. [64]	Jump based aquatic exercise	Fitness Center	RPE: 9–10	30 s	30 s	1:1	20	24	3	
Adamson et al. [80]	Cycle sprints	University	Max	6 s	60 s	1:10	6	8	1	
	Cycle sprints	University	Max	6 s	60 s	1:10	6	8	2	
Adamson et al. [85]	Cycle sprints	University	Max	6 s	60 s	1:10	6	10	2	
Angadi et al. [53]	Treadmill training	Lab	80–85% peak HR	4 min	3 min	4:3	4	4	3	
Ballesta-García et al. [76]	Cycling	NA	RPE: 12–18	4 min	2.5 min	3:5	NA	18	2	
Ballin et al. [75]	NA	Lab	RPE: 7/10	40 s	20 s	2:1	18	10	3	
Boa Sorte Silva et al. [25]	4 bouts of different exercise	Fitness Centre	80–95% max HR	4 min	3 min	4:3	4	24	3	
Boukabous et al. [23]	Treadmill	Lab	90% HRR	1 min	2 min	1:2	6	8	3	
Brown et al. [81]	Cycling	Lab	RPE:18, > 80% VO_2peak_	1 min	2 min	1:2	11	24	2	
Bruseghini et al. [67]	Cycling	Lab	85–95% VO_2max_	2 min	2 min	1:1	7	8	3	
Coetsee and Terblanche [71]	Treadmill	Lab	90–95% max HR	4 min	3 min	4:3	4	16	3	
Coswig et al. [14]	Treadmill	Healthcare Facilities	85–95% max HR	4 min	4 min	1:1	4	8	2	
Currie et al. [22]	Cycling	Healthcare Facilities	80–110% PPO	1 min	1 min	1:1	10	12	2	
de Matos et al. [58]	Treadmill	NA	85–90% max HR	1 min	1 min	1:1	10	12	2	
Fu et al. [52]	Cycling	Healthcare Facilities	80% HRR	3 min	3 min	1:1	5	12	3	
Herrod et al. [77]	Cycling	NA	90–110% PPO	1 min	1.5 min	2:3	4	6	3	
Hurst et al. [72]	Resistance exercises	Lab	> 90% max HR	4 min	3 min	4:3	4	12	2	
Hwang et al. [68]	Treadmill	Lab	90% peak HR	3 min	3 min	1:1	4	8	4	
Hwang et al. [59]	Treadmill	Lab	90% peak HR	3 min	3 min	1:1	4	8	4	
Iellamo et al. [57]	Treadmill	Healthcare Facilities	75–80% HR	4 min	3 min	4:3	4	12	3	
Izadi et al. [60]	Cycling	NA	85–90% of HRR	1.5 min	2 min	3:4	10	6	3	
Jiménez-García et al. [73]	Resistance exercises	Lab	90–95% max HR	4 min	3 min	4:3	4	12	2	
Kim et al. [74]	Cycling	Lab	90% peak HR	4 min	3 min	4:3	4	8	4	
Klonizakis et al. [66]	Cycling	Lab	100% PPO	1 min	1 min	1:1	10	2	1	
Kovacevic et al. [26]	Treadmill	Lab	RPE:16–18, 90–95% peak HR	4 min	3 min	4:3	4	12	3	
Krawcyk et al. [61]	Aerobic exercise	Home	RPE	3 min	2 min	3:2	3	12	5	
Li et al. [69]	Cycling	Lab	90% VO_2max_	3 min	3 min	1:1	4	12	3	
Maillard et al. [86]	Cycling	Lab	77–85% HR	8 s	12 s	2:3	15	16	2	
Mekari et al. [62]	Cycling	Lab	100% PPO	15 s	15 s	1:1	40	6	3	
Nilsson et al. [24]	Aerobic exercise	NA	RPE: 15–18	NA	NA	NA	NA	16	2	
Northey et al. [82]	Cycling	Lab	90% VO_2max_	30 s	2 min	1:4	7	12	3	
Nunes et al. [65]	Step climbing, free body weight squats	University	PRE: >16, > 80% PPO	1 min	1 min	1:1	10	12	3	
O’Brien et al. [63]	Cycling	Lab	100% PPO	15 s	15 s	1:1	40	6	3	
Reed et al. [55]	Cycling	Lab	80-100% PPO	30 s	30 s	1:1	8	12	2	
Rognmo et al. [56]	Treadmill	Lab	80–90% VO_2peak_	4 min	3 min	4:3	4	10	3	
Santos et al. [87]	Mixed	NA	RPE: 14–16	NA	30 s	5:12	NA	12	3	
Sculthorpe et al. [107]	Cycling	Lab	> 90% HRR	30 s	3 min	1:6	6	6	5	
Sian et al. [78]	Mixed	Lab	85% max HR	1 min	1.5 min	2:3	5	4	1	
	Mixed	Home	85% max HR	1 min	1.5 min	2:3	5	4	1	
Simonsson et al. [79]	Cycling	Lab	RPE: >= 16; 95% peak HR	6 s	54 s	1:9	10	10	2	
Spee et al. [54]	Cycling	Healthcare Facilities	85–95% VO_2peak_	4 min	3 min	4:3	4	12	3	
Terada et al. [83]	Cycling and treadmill	Fitness Center	100% VO_2_	1 min	3 min	1:3	11	12	5	
Varga et al. [84]	Cycling	Fitness Center	90% PPO	2 min	1 min	2:1	10	8	3	
Wisløff et al. [51]	Treadmill	Lab	90–95% peak HR	4 min	3 min	4:3	4	12	3	
Wyckelsma et al. [70]	Cycling	Lab	90–95% peak HR	4 min	4 min	1:1	4	12	3	

*Note*: RPE = rate of perceived exertion, HR = heart rate, HRR = heart rate reserve, VO_2peak_ = peak oxygen uptake, PPO = peak power output, VO_2max_ = maximum oxygen uptake, Lab = laboratory, max = maximum

**Table 3:** Summary of meta-analysis findings

Variable			Effect size				Test of heterogeneity				
			k	g	*P*	95%CI	Q	df	Tau^2^	I^2^	*P*
Resting heart rate											
	HR	HIIT vs. CON^1^	6	**-0.358**	0.032	[-0.671, -0.045]	3.90	5	0.00	0.00%	0.565
		HIIT vs. Active^2^	16	**-0.113**	0.029	[-0.213, -0.014]	4.18	15	0.00	0.00%	0.997
Resting blood pressure											
	SBP	HIIT vs. CON^1^	11	**-0.287**	0.008	[-0. 542, -0.032]	6.39	10	0.00	0.00%	0.782
		HIIT vs. Active^2^	20	**-0.143**	0.038	[-0.277, -0.009]	13.40	19	0.01	0.00%	0.817
	DBP	HIIT vs. CON^1^	9	-0.249	0.147	[-0.606, 0.108]	12.52	8	0.09	36.10%	0.129
		HIIT vs. Active^2^	18	-0.022	0.762	[-0.172, 0.128]	13.76	17	0.01	0.00%	0.684
Cardiorespiratory fitness											
	CRF	HIIT vs. CON^1^	16	**0.774**	0.000	[0.506, 1.041]	20.44	15	0.07	26.60%	0.156
		HIIT vs. Active^2^	21	**0.228**	0.008	[0.067, 0.383]	17.26	20	0.00	0.00%	0.636
Body composition											
	BMI	HIIT vs. CON^1^	13	-0.127	0.069	[-0.266, 0.014]	4.16	12	0.00	0.00%	0.980
		HIIT vs. Active^2^	16	-0.086	0.138	[-0.202, 0.031]	5.28	15	0.00	0.00%	0.989
	BF%	HIIT vs. CON^1^	7	**-0.257**	0.006	[-0.406, -0.108]	1.32	6	0.00	0.00%	0.970
		HIIT vs. Active^2^	12	-0.064	0.278	[-0.186, 0.059]	3.59	11	0.00	0.00%	0.980
	WC	HIIT vs. CON^1^	3	-0.155	0.161	[-0.462, 0.152]	1.29	6	0.00	0.00%	0.973
		HIIT vs. Active^2^	8	0.027	0.517	[-0.066, 0.120]	0.73	7	0.00	0.00%	0.998
Muscle fitness and mobility											
	MS	HIIT vs. CON^1^	6	**0.469**	0.004	[0.225, 0.713]	1.76	5	0.00	0.00%	0.882
		HIIT vs. Active^2^	8	**0.272**	0.033	[0.284, 0.516]	3.94	7	0.00	0.00%	0.787
	ME	HIIT vs. CON^1^	3	**0.648**	0.036	[0.101, 1.194]	0.53	2	0.00	0.00%	0.766
		HIIT vs. Active^2^	5	0.158	0.131	[-0.074, 0.389]	0.81	4	0.00	0.00%	0.938
	Balance	HIIT vs. CON^1^	6	**-0.794**	0.035	[-1.187, -0.401]	3.15	5	0.00	0.00%	0.677
		HIIT vs. Active^2^	5	0.008	0.977	[-0.724, 0.741]	6.87	4	0.12	41.80%	0.143

*Note*. ^1^Non-exercise condition; ^2^Other-exercise condition; HR = heart rate; SBP = systolic blood pressure; DBP = diastolic blood pressure; CRF = cardiorespiratory fitness; BMI = body mass index; BF% = body fat percentage; WC = waist circumference; MS = muscular strength; ME = muscular endurance; CI = confidence interval

Bold = result was significant

**Table 4:** Summary of publication bias and quality of evidence synthesis

Outcome	Summary of findings			Egger test			Quality of evidence synthesis (GRADE)						
	*n*	Sample size	I^2^	Intercept	t	*P*	Imprecision	Inconsistency	Indirectness	Risk of bias	Publication bias		Overall quality
**HIIT vs. non-exercise condition**													
HR	6	217	0.00%	-0.82	-0.72	0.51	-1	None	None	None	None		Moderate
SBP	10	340	0.00%	-1.61	-2.14	**0.06**	-1	None	None	None	-1		Low
DBP	9	291	36.10%	0.84	-0.57	0.58	-1	None	None	None	None		Moderate
CRF	15	413	26.60%	-0.75	-0.55	0.59	-1	None	None	None	None		Moderate
BMI	13	454	0.00%	0.64	0.93	0.37	-1	None	None	None	None		Moderate
BF%	7	241	0.00%	-0.49	0.83	0.44	None	None	None	None	None		High
WC	3	108	0.00%	-0.18	0.97	0.38	-1	None	None	None	None		Moderate
MS	4	115	0.00%	0.11	0.43	0.69	-1	None	None	None	None		Moderate
ME	2	81	0.00%	0.44	0.83	0.56	-1	None	None	None	None		Moderate
Balance	6	125	0.00%	4.13	1.36	0.25	-1	None	None	None	None		Moderate
**HIIT vs. other-exercise condition**													
HR	13	478	0.00%	-0.16	0.24	0.81	None	None	None	None	None		High
SBP	17	693	0.00%	-0.20	0.23	0.82	None	None	None	None	None		High
DBP	15	621	0.00%	0.10	-0.44	0.67	-1	None	None	None	None		Moderate
CRF	19	572	0.00%	-0.18	1.31	0.21	None	None	None	None	None		High
BMI	15	479	0.00%	-0.61	0.35	0.73	-1	None	None	None	None		Moderate
BF%	11	410	0.00%	-0.03	-0.16	0.88	-1	None	None	None	None		Moderate
WC	8	271	0.00%	0.14	-0.85	0.43	-1	None	None	None	None	Moderate	
MS	5	165	0.00%	1.12	-2.37	**0.06**	-1	None	None	None	-1	Low	
ME	4	124	0.00%	1.51	0.94	0.42	-1	None	None	None	None	Moderate	
Balance	4	104	41.80%	-2.13	0.84	0.46	-1	None	None	None	None	Moderate	

*Note*. *n* = the number of studies included in the analysis; HR = heart rate, SBP = systolic blood pressure, DBP = diastolic blood pressure, CRF = cardiorespiratory fitness, BMI = body mass index, BF% = body fat percentage, WC = waist circumference, MS = muscular strength, ME = muscular endurance

Bold = result was significant

In terms of body composition, we found that HIIT significantly improved BF% compared with no-intervention controls yet led to no significant changes in BMI and WC. This finding is echoed by a recent systematic review and meta-analysis conducted by Wu et al. [28], which reported a large effect of HIIT on BF% (WMD = -0.97) but no significant change in BMI in older adults compared with the control group. Regarding WC, an umbrella review reported that HIIT caused no significant improvement in this parameter across the lifespan [108]. A systematic review and meta-analysis by Batacan et al. also suggested that HIIT is an effective approach for reducing BF% in overweight or obese populations [99]. A possible explanation for this result might be HIIT induced the increased activity of catecholamines, which contributes to enhancing fat oxidation and releasing fat from visceral fat storage [109, 110]. Another possible explanation for this is the development of an elevated fat loss state due to decreased appetite and increased lipid metabolism for exercise recovery after the HIIT intervention [111, 112]. Although an observed decline in BF% was identified, there was no noticeable change in BMI, which may be explained by the gain in muscle mass [99]. HIIT appears to be effective in reducing body fat while simultaneously increasing muscle mass, likely due to multiple factors involving a range of physiological adaptations beyond just the afterburn effect (excess post-exercise oxygen consumption or EPOC) [113, 114].

The TUG test is a widely used tool for assessing functional balance in fall risk assessments [115]. It is recommended by the American Geriatrics Society and the British Geriatric Society as an instrument for measuring fall risk [116]. The present review found that HIIT had a moderate yet significant effect on balance compared with no-intervention controls, which is in line with previous studies [28, 30]. Notably, four studies in this review measured the TUG test outcome and one study measured the one-leg stand time (OLST). Two of these studies applied sprint interval training as the HIIT protocol [80, 85], and three studies conducted HIIT with jump-based aquatic exercise [64], cycling [69], or treadmill walking [71]. The dynamic balance and lower limb muscle strength resulting from the HIIT intervention may explain the improvement of balance capacity observed among older adults [117]. Additionally, HIIT had a significant small effect on MS compared with other-exercise intervention and no-intervention controls, which is in line with previous studies [28, 30]. These improvements may be due to the high-intensity nature of the exercise, which places greater demands on the muscles and can lead to adaptations such as increased muscle fibre recruitment, improved neuromuscular function, and increased muscle hypertrophy [79]. In comparison, other-exercise interventions typically engaged in low-to-moderate intensity exercise such as walking or cycling [14, 23, 79, 87], which may not provide the same level of stimulus to the muscles as HIIT. Thus, HIIT showed better benefits for MS. Regarding ME, HIIT showed nonsignificant overall effects compared with other-exercise interventions. These results are in line with those obtained in the study by Stern et al. [30] involving older adults aged 50 years and above, which concluded that the effect of HIIT vs. MICT on the STS was nonsignificant. A potential limitation to interpreting this result is that the intensities of HIIT and MICT protocols were inconsistent. One study [23] used low-volume HIIT (i.e., six sessions of 1-min intervals at 90% HRR with 2-min active recovery at 40% HRR) in comparison with MICT at 55%HRR for 50 min; another study [14] applied formal HIIT with four sets of 4-min intervals at 85-95% HR_max_ with 4-min recovery at 65% HR_max_ compared with MICT at 55-75%HR_max_ for 30 min. It remains unclear whether it is necessary to equalise energy expenditure or workload in comparative studies because of the possible differences in protocol intensities and programming [53]. We did not conduct meta-analyses of evidence on MP and flexibility due to the limited number of relevant studies. Regarding MP, two studies showed small beneficial effects of HIIT on leg power [72] and stair climb power [85] compared with no-intervention controls. Regarding flexibility, HIIT showed significant improvement when compared with no-intervention controls [69], but the difference was nonsignificant when compared with MICT [23, 69]. Overall, it is possible to improve balance and MS in older adults through HIIT.

### Limitations and Future Directions

Several limitations that could impact the results of this meta-analysis must be taken into consideration. (1) The GRADE quality of evidence reported in this study was generally low to moderate, and two potential factors caused these ratings to constrain the results’ certainty. First, the quality of the included studies was mixed. Most studies were at low risk of bias or had some concerns regarding quality (38/44) and had relatively small sample sizes. Therefore, the results should be interpreted with caution. Additional studies, particularly high-quality RCTs, are required to confirm out results. Second, given the generally low quality of grey literature (e.g., theses or dissertations), we did not include this type of literature, which might have caused potential publication bias [118]. In future reviews, diverse types of literature should be included to obtain more robust findings. (2) The results of meta-regression analyses of SBP and resting HR seemed to contradict common expectations. The underlying reasons for this are complex and should be further disentangled in future studies. They may relate to the quality of the included studies, including heterogeneity, and the difference in HIIT protocols. Future research can further explore the reasons for the contradiction. (3) Although we split the sample size of the shared group [78, 80] to address the unit-of-analysis problem during meta-analysis, the calculated effect sizes remained correlated [119]. Future studies should use advanced approaches (e.g. three-level meta-analysis model) to address this problem [119]. (4) Despite the inclusion of diverse indicators, some outcomes were rarely reported, namely MP, ME, and flexibility; therefore, the impact of HIIT on these outcomes remains unclear. Further high-quality studies should be implemented with a focus on the influence of HIIT on these outcomes.

The findings of our review suggest that HIIT, irrespective of sex and health status, can potentially improve physical fitness and health (e.g., resting HR, resting BP, CRF) in older adults. More specifically, our findings suggest that there are similar HIIT training-induced outcomes, irrespective of sex (male ratio), health status (clinical, non-clinical), duration (< 12 weeks, ≥ 12 weeks), frequency (< 3 times/weeks, ≥ 3 times/weeks), exercise mode (cycling, treadmill, others) and recovery mode (active, passive). Though there was still much variation in HIIT protocols, some components appeared in the literature more often, e.g. frequency is usually 2 times/week or 3 times/week; duration is usually 8 weeks or 12 weeks; exercise mode is usually cycling or treadmill mode; and WRR is usually 1:1 or 4:3. Future studies should compare these and other protocols for outcomes related to physical fitness and health as well as feasibility and tolerability.

## Conclusion

The significance of health-related physical fitness for older adults stems from its potential to uphold their physical independence, reduce the risk of chronic diseases, foster mental well-being, and augment social connections. The current systematic review and meta-analysis showed that HIIT may serve as a more efficacious intervention than other-exercise interventions or no-intervention controls in enhancing older adults’ physical fitness and health-related indicators, particularly in terms of improving their resting HR, SBP, CRF, BF%, MS, ME and balance. Our meta-regression analysis suggests that age may be a significant moderator for HR and SBP when compared to a non-exercise control group. Overall, this review demonstrates the need for more high-quality empirical studies specifically focused on understanding how participant characteristics, such as age, influence the effectiveness of HIIT interventions. Further investigation in this area could help optimize HIIT protocols to achieve the greatest benefits for older adults.

## Electronic Supplementary Material

Below is the link to the electronic supplementary material.

Supplementary Material 1

Supplementary Material 2

Supplementary Material 3

Supplementary Material 4

Supplementary Material 5

## Data Availability

The datasets used and/or analyses during the current study are available from the corresponding authors on reasonable request.

## Abbreviations

WHO: World Health Organization
ACSM: American College of Sport Medicine
CRF: Cardiorespiratory fitness
BMI: Body mass index
BF%: Body fat percent
WC: Waist circumference
ME: Muscular endurance
MS: Muscular strength
MP: Muscular power
HR: Heart rate
BP: Blood pressure
MICT: Moderate-intensity continuous training
HIIT: High-intensity interval training
HR_max_: Maximum heart rate
STS: Sit-to-stand test
TUG: Timed up and go
SMD: Standardized mean difference
6MWT: 6-min walking test
RCTs: Randomized controlled trials
SBP: Systolic blood pressure
DBP: Diastolic blood pressure
PRISMA: Preferred Reporting Items for Systematic reviews and Meta-Analyses
VO_2_: Oxygen uptake
HRR: Heart rate reserve
VO_2max_: Maximum oxygen uptake
VO_2peak_: Peak oxygen uptake
RoB2: Version 2 of the Cochrane Risk of Bias Tool
GRADE: Grading of Recommendations Assessment, Development and Evaluation
CIs: Confidence intervals
SD: Standard deviation
Corr: Correlation coefficient
RT: Resistance training
CT: Combined training
HICT: High-intensity continuous training
WMD: Weighted mean difference
MD: Mean difference
NO: Nitric oxide
EPOC: Excess post-exercise oxygen consumption
OLST: One-leg stand time
ECG: Electrocardiogram
PPG: Photoplethysmogram
