# Supplementary material for: Effects of High-Intensity Interval Training on the Parameters Related to Physical Fitness and Health of Older Adults: A Systematic Review and Meta-Analysis
Source: Sports Med Open. 2024 Sep 12;10:98. doi: 10.1186/s40798-024-00767-9 (PMC11393274; doi:10.1186/s40798-024-00767-9)
Supplement: Supplementary file 2 — Supplementary Material 2 [file 40798_2024_767_MOESM2_ESM.pdf]

Supplementary file 1

#1: (HIIT OR HIIE OR SIT OR high-intensity intermittent exercise OR high-intensity intermittent training OR high-intensity interval training OR high-intensity physical activity OR high-intensity interval exercise OR high intensity interval training OR high intensity interval exercise OR high intensity intermittent training OR high intensity physical activity OR high-intensity circuit training OR high intensity intermittent exercise OR high intensity training OR high-intensity exercise OR high-intensity training OR high intensity exercise OR high intensity circuit training OR high intensity aerobic interval training OR high aerobic intensity OR aerobic interval training OR aerobic interval exercise OR aerobic high intensity OR intermittent exercise OR intermittent training OR interval exercise OR interval training OR repeated sprint training OR sprint interval exercise OR sprint interval training)

#2: (physical fitness OR resting blood pressure OR “resting BP” OR resting heart rate OR “resting HR” OR body composition OR body mass index OR BMI OR circumference OR waist circumference OR hip circumference OR “waist-to-hip ratio” OR fat percentage OR “fat %” OR skinfold measurement OR densitometry OR cardiorespiratory fitness OR maximal oxygen uptake OR maximal oxygen consumption OR aerobic capacity OR “VO2max” OR “VO2peak” OR maximal aerobic velocity OR maximal aerobic speed OR cycle ergometer test OR treadmill test OR muscular fitness OR muscular strength OR chair stand test OR biceps curl test OR muscular endurance OR “6-minute walk test” OR 6MWT OR muscular power OR flexibility OR extension OR flexion OR “chair sit and reach test” OR motion test OR back scratch test OR balance OR Y-balance test OR balance error scoring system OR BESS OR “Up-and-Go test”)

#3: (intervention OR random* OR controlled OR trial* OR RCT OR randomized controlled trial OR randomised controlled trial OR CRCT OR C-RCT OR cluster randomized controlled trial OR cluster-randomized controlled trial OR cluster randomised controlled trial OR cluster-randomised controlled trial)

#4: (older adult* OR adult* OR elderly OR elders OR aged OR aging OR ageing OR senior*)

#1 AND #2 AND #3 AND #4
