# Supplementary material for: Effects of High-Intensity Interval Training on the Parameters Related to Physical Fitness and Health of Older Adults: A Systematic Review and Meta-Analysis
Source: Sports Med Open. 2024 Sep 12;10:98. doi: 10.1186/s40798-024-00767-9 (PMC11393274; doi:10.1186/s40798-024-00767-9)
Supplement: Supplementary file 3 — Supplementary Material 3 [file 40798_2024_767_MOESM3_ESM.pdf]

Supplementary file 3

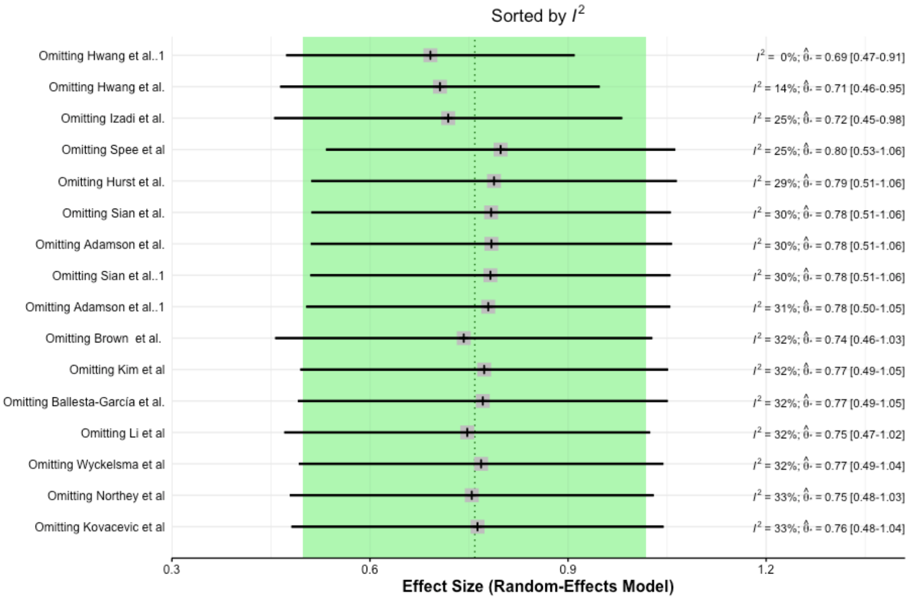

Fig. S1 Sensitivity test (cardiovascular fitness: HIIT vs non-exercise condition)

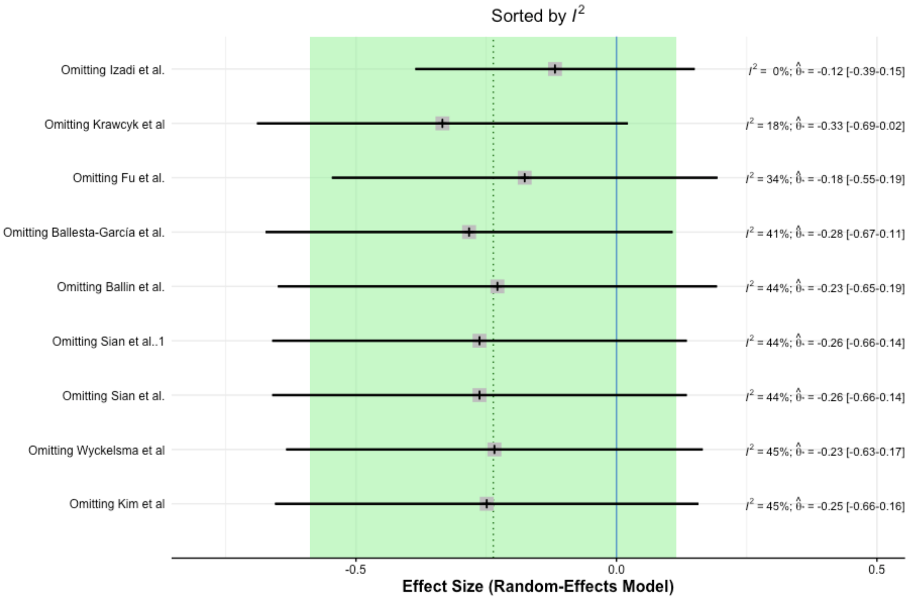

Fig. S2 Sensitivity test (diastolic blood pressure: HIIT vs non-exercise condition)

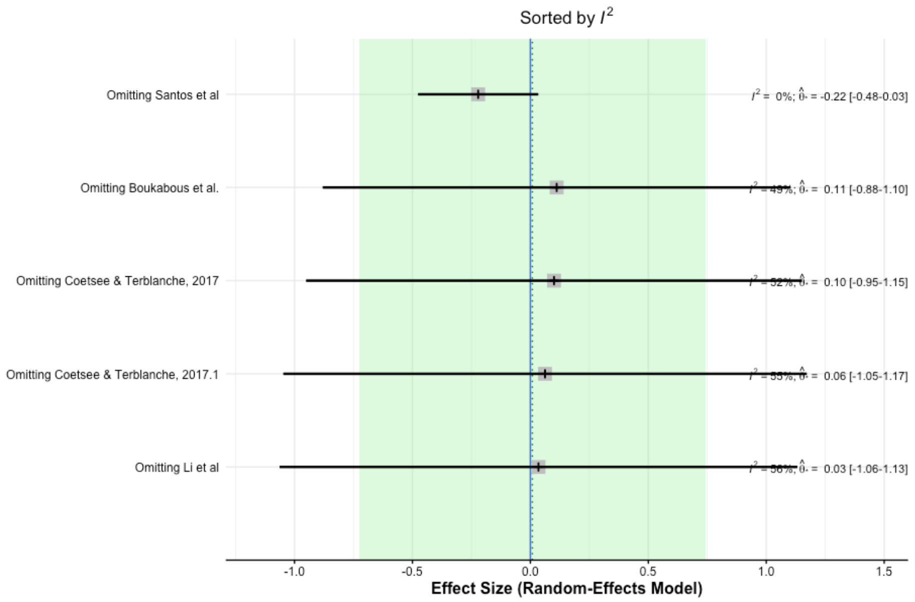

Fig. S3 Sensitivity test (balance: HIIT vs other-exercise condition)

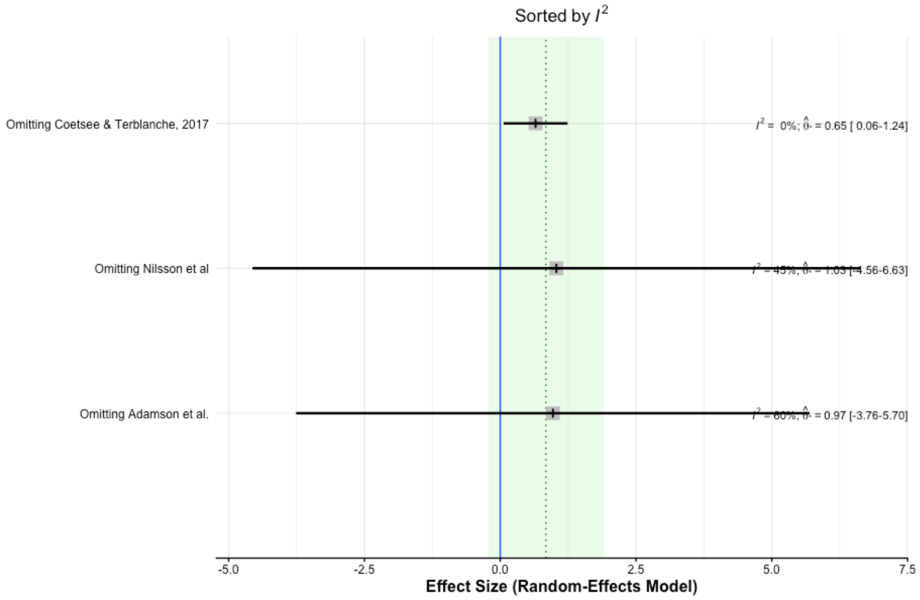

Fig. S4 Sensitivity test (muscular endurance HIIT vs non-exercise condition)
