# Supplementary material for: Effects of High-Intensity Interval Training on the Parameters Related to Physical Fitness and Health of Older Adults: A Systematic Review and Meta-Analysis
Source: Sports Med Open. 2024 Sep 12;10:98. doi: 10.1186/s40798-024-00767-9 (PMC11393274; doi:10.1186/s40798-024-00767-9)
Supplement: Supplementary file 4 — Supplementary Material 4 [file 40798_2024_767_MOESM4_ESM.pdf]

Supplementary file 2

Table S1. The summary of meta-regression findings

Category	Moderator	*k*	Estimate	*P*	95%CI	Test Statistic
Resting HR (HIIT vs other-exercise condition)						
Mean age	Intercept	16	1.346	0.021	[0.235, 2.457]	$t_{(1, 14)} = 7.981$*
	Year		-0.021	0.014	[-0.037, -0.005]	
Gender	Intercept	16	0.248	0.008	[-0.419, -0.077]	$t_{(1, 14)} = 4.023$
	Male(%)		0.302	0.065	[-0.021, 0.624]	
Health status	Non-clinical	9	-0.210	0.014	[-0.370; -0.050]	$t_{(1, 14)} = 2.607$
	Clinical	7	-0.060	0.129	[-0.049, 0.349]	
Attrition rate	Intercept	16	-0.167	0.026	[-0.311, -0.023]	$t_{(1, 14)} = 1.237$
	Attrition (%)		0.005	0.285	[-0.005, 0.015]	
Duration	< 12 weeks	8	-0.127	0.120	[-0.290, 0.037]	$t_{(1, 14)} = 0.049$
	≥ 12 weeks	8	-0.104	0.828	[-0.190, 0.234]	
Frequency	< 3times/week	6	-0.194	0.015	[-0.344, -0.045]	$t_{(1, 14)} = 2.292$
	≥ 3times/week	10	-0.056	0.152	[-0.058, 0.334]	
Exercise	Cycling	7	-0.086	0.167	[-0.212, 0.041]	$t_{(2, 12)} = 0.836$
	Treadmill	7	-0.196	0.231	[-0.302, 0.081]	
	Other	1	-0.166	0.505	[-0.336, 0.175]	
Recovery mode	Active	12	-0.184	0.001	[-0.276, -0.092]	$t_{(1, 13)} = 3.971$
	Passive	3	-0.025	0.068	[-0.182, 0.132]	
SBP (HIIT vs other-exercise condition)						
Mean age	Intercept	20	-0.936	0.349	[-2.981, 1.109]	$t_{(1, 18)} = 0.669$
	Year		0.012	0.424	[-0.018, 0.041]	
Gender	Intercept	20	-0.235	0.078	[-0.498, 0.029]	$t_{(1, 18)} = 0.754$
	Male(%)		0.223	0.397	[-0.317, 0.763]	
Health status	Non-clinical	11	-0.188	0.047	[-0.373, -0.003]	$t_{(1, 18)} = 0.607$
	Clinical	9	-0.086	0.446	[-0.173, 0.377]	
Attrition rate	Intercept	20	-0.170	0.113	[-0.385, 0.045]	$t_{(1, 18)} = 0.134$
	Attrition (%)		0.003	0.719	[-0.013, 0.019]	
Duration	< 12 weeks	11	-0.146	0.186	[-0.368, 0.077]	$t_{(1, 18)} = 0.002$
	≥ 12 weeks	9	-0.14	0.966	[-0.279, 0.291]	
Frequency	< 3times/week	9	-0.165	0.084	[-0.355, 0.024]	$t_{(1, 18)} = 0.138$
	≥ 3times/week	11	-0.116	0.714	[-0.228, 0.326]	
Exercise	Cycling	10	-0.001	0.949	[-0.183, 0.172]	$t_{(2, 17)} = 2.866$
	Treadmill	9	-0.295	0.029	[-0.566, -0.036]	
	Other	1	-0.148	0.479	[-0.557, 0.273]	
Recovery mode	Active	15	-0.222	0.007	[-0.376, -0.068]	$t_{(2, 17)} = 2.036$
	Passive	4	0.040	0.089	[-0.459, 0.539]	
	Both	1	0.047	0.257	[-0.487, 0.580]	
SBP (HIIT vs non-exercise condition)						
Mean age	Intercept	11	-2.664	0.027	[-4.942, -0.386]	$t_{(1, 9)} = 5.600$*

	Year		0.034	0.042	[0.002, 0.067]	
Gender	Intercept	11	-0.157	0.569	[-0.756, 0.443]	$t_{(1, 9)} = 0.274$
	Male(%)		-0.263	0.614	[-1.399, 0.873]	
Health status	Non-clinical	5	-0.402	0.007	[-0.664, -0.141]	$t_{(1, 9)} = 2.044$
	Clinical	6	0.237	0.187	[-0.543, 0.147]	
Attrition rate	Intercept	11	-0.198	0.226	[-0.478, 0.109]	$t_{(1, 9)} = 0.516$
	Attrition (%)		-0.010	-0.007	[-0.031, 0.016]	
Duration	< 12 weeks	7	-0.343	0.014	[-0.599, -0.087]	$t_{(1, 9)} = 0.637$
	≥ 12 weeks	4	0.145	0.445	[-0.266, 0.557]	
Frequency	< 3times/week	3	-0.203	0.394	[-0.716, 0.310]	$t_{(1, 9)} = 0.164$
	≥ 3times/week	8	-0.100	0.695	[-0.660, 0.459]	
Exercise	Cycling	7	-0.316	0.033	[-0.599, -0.032]	$t_{(2, 12)} = 0.083$
	Treadmill	1	0.031	0.928	[-0.412, 0.589]	
	Other	3	0.088	0.695	[-0.412, 0.589]	
Recovery mode	Active	10	-0.318	0.012	[-0.546, -0.090]	$t_{(1, 9)} = 0.442$
	Passive	1	0.146	0.523	[-0.350, 0.641]	
CRF (HIIT vs other-exercise condition)						
Mean age	Intercept	21	1.278	0.451	[-2.200, 4.756]	$t_{(1, 19)} = 0.400$
	Year		-0.016	0.535	[-0.068, 0.037]	
Gender	Intercept	21	0.260	0.164	[-0.116, 0.636]	$t_{(1, 19)} = 0.040$
	Male(%)		-0.058	0.845	[-0.663, 0.548]	
Health status	Non-clinical	13	0.208	0.037	[0.014, 0.402]	$t_{(1, 19)} = 0.117$
	Clinical	8	0.072	0.688	[-0.297, 0.440]	
Attrition rate	Intercept	21	0.116	0.274	[-0.099, 0.330]	$t_{(1, 19)} = 2.542$
	Attrition (%)		0.016	0.127	[-0.005, 0.037]	
Duration	< 12 weeks	12	0.247	0.037	[0.017, 0.477]	$t_{(1, 19)} = 0.400$
	≥ 12 weeks	9	-0.039	0.807	[-0.370, 0.292]	
Frequency	< 3times/week	4	0.166	0.276	[-0.144, 0.477]	$t_{(1, 19)} = 0.400$
	≥ 3times/week	17	0.086	0.628	[-0.280, 0.452]	
Exercise	Cycling	13	0.187	0.068	[-0.016, 0.339]	$t_{(2, 18)} = 0.320$
	Treadmill	7	0.329	0.434	[-0.232, 0.517]	
	Other	1	0.232	0.931	[-1.024, 1.114]	
Recovery mode	Active	16	0.286	0.005	[0.097, 0.476]	$t_{(2, 18)} = 0.835$
	Passive	4	0.088	0.379	[-0.186, 0.362]	
	Both	1	0.017	0.310	[-0.490, 0.523]	
CRF (HIIT vs non-exercise condition)						
Mean age	Intercept	16	4.607	0.074	[-0.508, 9.723]	$t_{(1, 14)} = 2.586$
	Year		-0.058	0.130	[-0.134, 0.019]	
Gender	Intercept	16	0.598	0.071	[-0.058, 1.254]	$t_{(1, 14)} = 0.408$
	Male(%)		0.387	0.518	[-0.866, 1.640]	
Health status	Non-clinical	11	0.663	0.000	[0.364, 0.963]	$t_{(1, 14)} = 2.402$
	Clinical	5	0.592	0.144	[-0.157, 0.972]	
Attrition rate	Intercept	14	0.474	0.012	[0.122, 0.827]	$t_{(1, 14)} = 5.973^{*}$

	Attrition (%)		0.027	0.028	[0.003, 0.050]	
Duration	< 12 weeks	8	0.946	0.000	[0.543, 1.350]	$t_{(1, 14)} = 1.377$
	≥ 12 weeks	8	-0.290	0.260	[-0.821, 0.240]	
Frequency	< 3times/week	7	0.555	0.005	[0.194, 0.915]	$t_{(1, 14)} = 3.335$
	≥ 3times/week	9	0.422	0.089	[-0.074, 0.916]	
Exercise	Cycling	9	0.992	0.000	[0.688, 1.297]	$t_{(2, 13)} = 2.984$
	Treadmill	1	-0.288	0.495	[-1.174, 0.598]	
	Other	6	-0.576	0.030	[-1.088, -0.065]	
Recovery mode	Active	13	0.866	0.000	[0.588, 1.144]	$t_{(1, 14)} = 2.426$
	Passive	3	-0.475	0.142	[-1.129, 0.179]	
BF% (HIIT vs non-exercise condition)						
Mean age	Intercept	7	-1.737	0.340	[-5.974, 2.499]	$t_{(1, 5)} = 0.808$
	Year		0.022	0.410	[-0.041, 0.085]	
Gender	Intercept	7	-0.196	0.210	[-0.547, 0.155]	$t_{(1, 5)} = 0.259$
	Male(%)		-0.125	0.633	[-0.755, 0.506]	
Health status	Non-clinical	4	-0.284	0.014	[-0.483, -0.085]	$t_{(1, 5)} = 0.396$
	Clinical	3	-0.196	0.557	[-0.270, 0.446]	
Attrition rate	Intercept	7	-0.173	0.139	[-0.425, 0.080]	$t_{(1, 5)} = 1.179$
	Attrition (%)		-0.010	0.327	[-0.034, 0.014]	
Duration	< 12 weeks	3	-0.316	0.035	[-0.599, -0.034]	$t_{(1, 5)} = 0.441$
	≥ 12 weeks	4	-0.226	0.536	[-0.258, 0.438]	
Frequency	< 3times/week	3	-0.218	0.051	[-0.438, 0.001]	$t_{(1, 5)} = 0.464$
	≥ 3times/week	4	-0.306	0.526	[-0.419, 0.243]	
Exercise	Cycling	4	-0.320	0.007	[-0.507, -0.134]	$t_{(1, 5)} = 1.955$
	Other	3	-0.158	0.221	[-0.471, 0.155]	
Recovery mode	Active	6	-0.250	0.016	[-0.429, -0.070]	$t_{(1, 5)} = 0.114$
	Passive	1	-0.324	0.749	[-0.641, 0.492]	

Note: HR = heart rate, SBP = systolic blood pressure, CRF = cardiorespiratory fitness, BF% = body fat percentage, HIIT = high-intensity interval training.
