# Supplementary material for: Effects of High-Intensity Interval Training on the Parameters Related to Physical Fitness and Health of Older Adults: A Systematic Review and Meta-Analysis
Source: Sports Med Open. 2024 Sep 12;10:98. doi: 10.1186/s40798-024-00767-9 (PMC11393274; doi:10.1186/s40798-024-00767-9)
Supplement: Supplementary file 5 — Supplementary Material 5 [file 40798_2024_767_MOESM5_ESM.pdf]

ID	Article	Exclusion Reasons				
		P	I	C	O	S
1	Ahmadizad, S., Nouri-Habashi, A., Rahmani, H., Maleki, M., Naderi, N., Lotfian, S., & Salimian, M. (2016). Platelet activation and function in response to high intensity interval exercise and moderate continuous exercise in CABG and PCI patients. Clinical hemorheology and microcirculation, 64(4), 911-919.	×				
2	Ahmed, U., Karimi, H., Amir, S., & Ahmed, A. (2021). Effects of intensive multiplanar trunk training coupled with dual-task exercises on balance, mobility, and fall risk in patients with stroke: a randomized controlled trial. Journal of international medical research, 49(11), 03000605211059413.		×			
3	Andonian, B. J., Bartlett, D. B., Huebner, J. L., Willis, L., Hoselton, A., Kraus, V. B., ... & Huffman, K. M. (2018). Effect of high-intensity interval training on muscle remodeling in rheumatoid arthritis compared to prediabetes. Arthritis research & therapy, 20, 1-9.			×		×
4	Askim, T., Dahl, A. E., Aamot, I. L., Hokstad, A., Helbostad, J., & Indredavik, B. (2014). High‐intensity aerobic interval training for patients 3–9 months after stroke. A feasibility study. Physiotherapy research international, 19(3), 129-139.			×		×
5	Bartlett, D. B., Willis, L. H., Slentz, C. A., Hoselton, A., Kelly, L., Huebner, J. L., ... & Huffman, K. M. (2018). Ten weeks of high-intensity interval walk training is associated with reduced disease activity and improved innate immune function in older adults with rheumatoid arthritis: a pilot study. Arthritis research & therapy, 20, 1-15.			×		×
6	Bell, K. E., Fang, H., Snijders, T., Allison, D. J., Zulyniak, M. A., Chabowski, A., ... & Heisz, J. J. (2019). A multi-ingredient nutritional supplement in combination with resistance exercise and high-intensity interval training improves cognitive function and increases N-3 index in healthy older men: a randomized controlled trial. Frontiers in Aging Neuroscience, 11, 107.		×			
7	Bell, K. E., Séguin, C., Parise, G., Baker, S. K., & Phillips, S. M. (2015). Day-to-day changes in muscle protein synthesis in recovery from resistance, aerobic, and high-intensity interval exercise in older men. Journals of Gerontology Series A: Biomedical Sciences and Medical Sciences, 70(8), 1024-1029.		×			
8	Benda, N. M., Seeger, J. P., Stevens, G. G., Hijmans-Kersten, B. T., van Dijk, A. P., Bellersen, L., ... & Thijssen, D. H. (2015). Effects of high-intensity interval training versus continuous training on physical fitness, cardiovascular function and quality of life in heart failure patients. PLoS One, 10(10), e0141256.			×		×

9	Benetti, M., Araujo, C. L. P. D., & Santos, R. Z. D. (2010). Cardiorespiratory fitness and quality of life at different exercise intensities after myocardial infarction. Arquivos brasileiros de cardiologia, 95, 399-404.	×				
10	Bertani, R. F., Campos, G. O., Perseguin, D. M., Bonardi, J. M., Ferriolli, E., Moriguti, J. C., & Lima, N. K. (2018). Resistance exercise training is more effective than interval aerobic training in reducing blood pressure during sleep in hypertensive elderly patients. The Journal of Strength & Conditioning Research, 32(7), 2085-2090.		×			
11	Boereboom, C. L., Phillips, B. E., Williams, J. P., & Lund, J. N. (2016). A 31-day time to surgery compliant exercise training programme improves aerobic health in the elderly. Techniques in coloproctology, 20, 375-382.			×		×
12	Bouaziz, W., Schmitt, E., Vogel, T., Lefebvre, F., Leprêtre, P. M., Kaltenbach, G., ... & Lang, P. O. (2019). Effects of a short-term Interval Aerobic Training Programme with active Recovery bouts (IATP-R) on cognitive and mental health, functional performance and quality of life: A randomised controlled trial in sedentary seniors. International journal of clinical practice, 73(1), e13219.		×			
13	Bouaziz, W., Schmitt, E., Vogel, T., Lefebvre, F., Remetter, R., Lonsdorfer, E., ... & Lang, P. O. (2018). Effects of Interval Aerobic Training Program with Recovery bouts on cardiorespiratory and endurance fitness in seniors. Scandinavian journal of medicine & science in sports, 28(11), 2284-2292.		×			
14	Briggs, B. C., Ryan, A. S., Sorkin, J. D., & Oursler, K. K. (2021). Feasibility and effects of high-intensity interval training in older adults living with HIV. Journal of sports sciences, 39(3), 304-311.		×			
15	Bruseghini, P., Calabria, E., Tam, E., Milanese, C., Oliboni, E., Pezzato, A., ... & Capelli, C. (2015). Effects of eight weeks of aerobic interval training and of isoinertial resistance training on risk factors of cardiometabolic diseases and exercise capacity in healthy elderly subjects. Oncotarget, 6(19), 16998.			×		×
16	Buckinx, F., Gaudreau, P., Marcangeli, V., Boutros, G. E. H., Dulac, M. C., Morais, J. A., & Aubertin-Leheudre, M. (2019). Muscle adaptation in response to a high-intensity interval training in obese older adults: effect of daily protein intake distribution. Aging clinical and experimental research, 31, 863-874.			×		×

17	Buckinx, F., Gouspillou, G., Carvalho, L. P., Marcangeli, V., El Hajj Boutros, G., Dulac, M., ... & Aubertin-Leheudre, M. (2018). Effect of high-intensity interval training combined with L-citrulline supplementation on functional capacities and muscle function in dynapenic-obese older adults. Journal of clinical medicine, 7(12), 561.		×			
18	Chien, K. Y., Kan, N. W., Liao, Y. H., Yang, W. T., & Yang, Y. (2020). Land vs. water HIIE effects on muscle oxygenation and physiological parameter responses in postmenopausal women. Scientific Reports, 10(1), 13754.			×		×
19	Chrøis, K. M., Dohlmann, T. L., Søgaard, D., Hansen, C. V., Dela, F., Helge, J. W., & Larsen, S. (2020). Mitochondrial adaptations to high intensity interval training in older females and males. European Journal of Sport Science, 20(1), 135-145.			×		×
20	Chrysohoou, C., Tsitsinakis, G., Vogiatzis, I., Cherouveim, E., Antoniou, C., Tsiantilas, A., ... & Stefanadis, C. (2014). High intensity, interval exercise improves quality of life of patients with chronic heart failure: a randomized controlled trial. QJM: An International Journal of Medicine, 107(1), 25-32.	×				
21	Craighead, D. H., Heinbockel, T. C., Hamilton, M. N., Bailey, E. F., MacDonald, M. J., Gibala, M. J., & Seals, D. R. (2019). Time-efficient physical training for enhancing cardiovascular function in midlife and older adults: promise and current research gaps. Journal of Applied Physiology, 127(5), 1427-1440.			×		×
22	Currie, K. D., Bailey, K. J., Jung, M. E., McKelvie, R. S., & MacDonald, M. J. (2015). Effects of resistance training combined with moderate-intensity endurance or low-volume high-intensity interval exercise on cardiovascular risk factors in patients with coronary artery disease. Journal of science and medicine in sport, 18(6), 637-642.		×			
23	de Castro Cezar, N. O., Ansai, J. H., de Oliveira, M. P. B., da Silva, D. C. P., de Lima Gomes, W., Barreiros, B. A., ... & de Andrade, L. P. (2021). Feasibility of improving strength and functioning and decreasing the risk of falls in older adults with Alzheimer's dementia: a randomized controlled home-based exercise trial. Archives of gerontology and geriatrics, 96, 104476. ISO 690		×			
24	Deka, P., Pathak, D., Klompstra, L., Sempere-Rubio, N., Querol-Giner, F., & Marques-Sule, E. (2022). High-intensity interval and resistance training improve health outcomes in older adults with coronary disease. Journal of the American Medical Directors Association, 23(1), 60-65.					×

25	Devin, J. L., Hill, M. M., Mourtzakis, M., Quadrilatero, J., Jenkins, D. G., & Skinner, T. L. (2019). Acute high intensity interval exercise reduces colon cancer cell growth. The Journal of physiology, 597(8), 2177-2184.			×		×
26	Donath, L., Kurz, E., Roth, R., Zahner, L., & Faude, O. (2015). Different ankle muscle coordination patterns and co-activation during quiet stance between young adults and seniors do not change after a bout of high intensity training. BMC geriatrics, 15, 1-8.			×		×
27	Hwang C-L, Yoo J-K, Kim H-K, Hwang M-H, Handberg EM, Petersen JW, et al. Novel all-extremity high-intensity interval training improves aerobic fitness, cardiac function and insulin resistance in healthy older adults. Exp Gerontol. 2016;82:112–9.			×		×
28	Gayda, M., Normandin, E., Meyer, P., Juneau, M., Haykowsky, M., & Nigam, A. (2012). Central hemodynamic responses during acute high-intensity interval exercise and moderate continuous exercise in patients with heart failure. Applied Physiology, Nutrition, and Metabolism, 37(6), 1171-1178.	×				
29	Gjellesvik, T. I., Becker, F., Tjønna, A. E., Indredavik, B., Lundgaard, E., Solbakken, H., ... & Askim, T. (2021). Effects of high-intensity interval training after stroke (the HIIT stroke study) on physical and cognitive function: a multicenter randomized controlled trial. Archives of physical medicine and rehabilitation, 102(9), 1683-1691.	×				
30	Grace, F., Herbert, P., Elliott, A. D., Richards, J., Beaumont, A., & Sculthorpe, N. F. (2018). High intensity interval training (HIIT) improves resting blood pressure, metabolic (MET) capacity and heart rate reserve without compromising cardiac function in sedentary aging men. Experimental Gerontology, 109, 75-81.			×		×
31	Guadalupe-Grau, A., Aznar-Laín, S., Mañas, A., Castellanos, J., Alcázar, J., Ara, I., ... & García-García, F. J. (2017). Short-and long-term effects of concurrent strength and HIIT training in octogenarians with COPD. Journal of Aging and Physical Activity, 25(1), 105-115.			×		×
32	Guiraud, T., Juneau, M., Nigam, A., Gayda, M., Meyer, P., Mekary, S., ... & Bosquet, L. (2010). Optimization of high intensity interval exercise in coronary heart disease. European journal of applied physiology, 108, 733-740.			×		×

33	Hayes, L. D., Herbert, P., Sculthorpe, N., & Grace, F. (2020). High intensity interval training (HIIT) produces small improvements in fasting glucose, insulin, and insulin resistance in sedentary older men but not masters athletes. Experimental Gerontology, 140, 111074.			×		×
34	Helgerud, J., Wang, E., Mosti, M. P., Wiggen, Ø. N., & Hoff, J. (2009). Plantar flexion training primes peripheral arterial disease patients for improvements in cardiac function. European journal of applied physiology, 106, 207-215.			×		×
35	Herbert, P., Hayes, L. D., Beaumont, A. J., Grace, F. M., & Sculthorpe, N. F. (2021). Six weeks of high intensity interval training (HIIT) facilitates a four year preservation of aerobic capacity in sedentary older males: A reunion study. Experimental Gerontology, 150, 111373.		×			
36	Herbert, P., Hayes, L. D., Sculthorpe, N. F., & Grace, F. M. (2017). HIIT produces increases in muscle power and free testosterone in male masters athletes. Endocrine connections, 6(7), 430-436.			×		×
37	Herbert, P., Hayes, L. D., Sculthorpe, N., & Grace, F. M. (2017). High-intensity interval training (HIIT) increases insulin-like growth factor-I (IGF-I) in sedentary aging men but not masters' athletes: an observational study. The Aging Male, 20(1), 54-59.			×		×
38	Hollekim-Strand, S. M., Bjørgaas, M. R., Albrektsen, G., Tjønna, A. E., Wisløff, U., & Ingul, C. B. (2014). High-intensity interval exercise effectively improves cardiac function in patients with type 2 diabetes mellitus and diastolic dysfunction: a randomized controlled trial. Journal of the American College of Cardiology, 64(16), 1758-1760.	×				
39	Hsu, C. C., Fu, T. C., Huang, S. C., Chen, C. P. C., & Wang, J. S. (2021). Increased serum brain-derived neurotrophic factor with high-intensity interval training in stroke patients: a randomized controlled trial. Annals of physical and rehabilitation medicine, 64(4), 101385.	×				
40	Huang, S. C., Wong, M. K., Lin, P. J., Tsai, F. C., Fu, T. C., Wen, M. S., ... & Wang, J. S. (2014). Modified high-intensity interval training increases peak cardiac power output in patients with heart failure. European journal of applied physiology, 114, 1853-1862.	×				

41	Hyun, S. J., Lee, J., & Lee, B. H. (2021). The effects of sit-to-stand training combined with real-time visual feedback on strength, balance, gait ability, and quality of life in patients with stroke: A randomized controlled trial. International Journal of Environmental Research and Public Health, 18(22), 12229.		×			
42	Isaksen, K., Munk, P. S., Giske, R., & Larsen, A. I. (2016). Effects of aerobic interval training on measures of anxiety, depression and quality of life in patients with ischaemic heart failure and an implantable cardioverter defibrillator: A prospective non-randomized trial. Journal of Rehabilitation Medicine, 48(3), 300-306.					×
43	Isaksen, K., Munk, P. S., Valborgland, T., & Larsen, A. I. (2015). Aerobic interval training in patients with heart failure and an implantable cardioverter defibrillator: a controlled study evaluating feasibility and effect. European journal of preventive cardiology, 22(3), 296-303.					×
44	Jabbour, G., & Majed, L. (2018). Ratings of perceived exertion misclassify intensities for sedentary older adults during graded cycling test: effect of supramaximal high-intensity interval training. Frontiers in physiology, 9, 410411.	×				
45	Jaureguizar, K. V., Vicente-Campos, D., Bautista, L. R., de la Peña, C. H., Gómez, M. J. A., Rueda, M. J. C., & Mahillo, I. F. (2016). Effect of high-intensity interval versus continuous exercise training on functional capacity and quality of life in patients with coronary artery disease: a randomized clinical trial. Journal of cardiopulmonary rehabilitation and prevention, 36(2), 96-105.	×				
46	Jürimäe, J., Purge, P., Remmel, L., Ereline, J., Kums, T., Kamandulis, S., ... & Pääsuke, M. (2022). Changes in irisin, inflammatory cytokines and aerobic capacity in response to three weeks of supervised sprint interval training in older men. The Journal of Sports Medicine and Physical Fitness.			×		×
47	Kamiya, K., Hayashi, E., Ito, Y., Kudo, A., Kakihana, H., Tsuda, K., ... & Tamaki, J. (2023). Effect of high-intensity interval walking on microvascular endothelial function among community-dwelling older people. Geriatrics & Gerontology International, 23(2), 103-110.			×		×
48	Keogh, J. W., Grigg, J., & Vertullo, C. J. (2018). Is high-intensity interval cycling feasible and more beneficial than continuous cycling for knee osteoarthritic patients? Results of a randomised control feasibility trial. PeerJ, 6, e4738.	×				

49	Knowles, A. M., Herbert, P., Easton, C., Sculthorpe, N., & Grace, F. M. (2015). Impact of low-volume, high-intensity interval training on maximal aerobic capacity, health-related quality of life and motivation to exercise in ageing men. Age, 37, 1-12.			×		×
50	Koufaki, P., Mercer, T. H., George, K. P., & Nolan, J. (2014). Low-volume high-intensity interval training vs continuous aerobic cycling in patients with chronic heart failure: a pragmatic randomised clinical trial of feasibility and effectiveness. Journal of rehabilitation medicine, 46(4), 348-356.	×				
51	Krusnauskas, R., Venckunas, T., Snieckus, A., Eimantas, N., Baranauskiene, N., Skurvydas, A., ... & Kamandulis, S. (2018). Very low volume high-intensity interval exercise is more effective in young than old women. BioMed Research International, 2018.			×		×
52	Linares, A. M., Goncin, N., Stuckey, M., Burgomaster, K. A., & Dogra, S. (2022). Acute Cardiopulmonary Response to Interval and Continuous Exercise in Older Adults: A Randomized Crossover Study. Journal of strength and conditioning research, 36(10), 2920–2926.					×
53	Lepretre, P. M., Vogel, T., Brechat, P. H., Dufour, S., Richard, R., Kaltenbach, G., ... & Lonsdorfer, J. (2009). Impact of short-term aerobic interval training on maximal exercise in sedentary aged subjects. International journal of clinical practice, 63(10), 1472-1478.			×		×
54	Losa-Reyna, J., Baltasar-Fernandez, I., Alcazar, J., Navarro-Cruz, R., Garcia-Garcia, F. J., Alegre, L. M., & Alfaro-Acha, A. (2019). Effect of a short multicomponent exercise intervention focused on muscle power in frail and pre frail elderly: a pilot trial. Experimental Gerontology, 115, 114-121.		×			
55	Masuki, S., Nishida, K., Hashimoto, S., Morikawa, M., Takasugi, S., Nagata, M., ... & Nose, H. (2017). Effects of milk product intake on thigh muscle strength and NFKB gene methylation during home-based interval walking training in older women: a randomized, controlled pilot study. PLoS One, 12(5), e0176757.		×			
56	Mejías-Peña, Y., Rodriguez-Miguelez, P., Fernandez-Gonzalo, R., Martínez-Flórez, S., Almar, M., de Paz, J. A., ... & González-Gallego, J. (2016). Effects of aerobic training on markers of autophagy in the elderly. Age, 38, 1-12.		×			

57	Moholdt, T., Madssen, E., Rognmo, Ø., & Aamot, I. L. (2014). The higher the better? Interval training intensity in coronary heart disease. Journal of science and medicine in sport, 17(5), 506-510.	×				
58	Molmen, H. E., Wisloff, U., Aamot, I. L., Stoylen, A., & Ingul, C. B. (2012). Aerobic interval training compensates age related decline in cardiac function. Scandinavian Cardiovascular Journal, 46(3), 163-171.			×		×
59	Morikawa, M., Nakano, S., Mitsui, N., Murasawa, H., Masuki, S., & Nose, H. (2018). Effects of dried tofu supplementation during interval walking training on the methylation of the NFKB2 gene in the whole blood of older women. The journal of physiological sciences, 68, 749-757.		×			
60	Moro, T., Tinsley, G., Bianco, A., Gottardi, A., Gottardi, G. B., Faggian, D., ... & Paoli, A. (2017). High intensity interval resistance training (HIIRT) in older adults: Effects on body composition, strength, anabolic hormones and blood lipids. Experimental gerontology, 98, 91-98.			×		×
61	Müller, D. C., Boeno, F. P., Izquierdo, M., Aagaard, P., Teodoro, J. L., Grazioli, R., ... & Cadore, E. L. (2021). Effects of high-intensity interval training combined with traditional strength or power training on functionality and physical fitness in healthy older men: A randomized controlled trial. Experimental Gerontology, 149, 111321.		×			
62	Munch, G. W., Iepsen, U. W., Ryrsø, C. K., Rosenmeier, J. B., Pedersen, B. K., & Mortensen, S. P. (2018). Effect of 6 wk of high-intensity one-legged cycling on functional sympatholysis and ATP signaling in patients with heart failure. American Journal of Physiology-Heart and Circulatory Physiology, 314(3), H616-H626.			×		×
63	Munk, P. S., Butt, N., & Larsen, A. I. (2010). High-intensity interval exercise training improves heart rate variability in patients following percutaneous coronary intervention for angina pectoris. International journal of cardiology, 145(2), 312-314.	×				
64	Munk, P. S., Staal, E. M., Butt, N., Isaksen, K., & Larsen, A. I. (2009). High-intensity interval training may reduce in-stent restenosis following percutaneous coronary intervention with stent implantation: a randomized controlled trial evaluating the relationship to endothelial function and inflammation. American heart journal, 158(5), 734-741.	×				

65	Nakajima, K., Takeoka, M., Mori, M., Hashimoto, S., Sakurai, A., Nose, H., ... & Taniguchi, S. (2010). Exercise effects on methylation of ASC gene. International journal of sports medicine, 671-675.			×		×
66	Negaresh, R., Motl, R., Mokhtarzade, M., Ranjbar, R., Majdinasab, N., Khodadoost, M., ... & Patel, D. (2019). Effect of short-term interval exercise training on fatigue, depression, and fitness in normal weight vs. overweight person with multiple sclerosis. Explore, 15(2), 134-141.		×			
67	Osuka, Y., Matsubara, M., Hamasaki, A., Hiramatsu, Y., Ohshima, H., & Tanaka, K. (2017). Development of low-volume, high-intensity, aerobic-type interval training for elderly Japanese men: a feasibility study. European Review of Aging and Physical Activity, 14, 1-8.			×		×
68	Pandey, A., Suskin, N., & Poirier, P. (2017). The impact of burst exercise on cardiometabolic status of patients newly diagnosed with type 2 diabetes. Canadian Journal of Cardiology, 33(12), 1645-1651.		×			
69	Parker, L., Trewin, A., Levinger, I., Shaw, C. S., & Stepto, N. K. (2017). The effect of exercise-intensity on skeletal muscle stress kinase and insulin protein signaling. PLoS One, 12(2), e0171613.	×				
70	Paschen, S., Hansen, C., Welzel, J., Albrecht, J., Atrsaei, A., Aminian, K., ... & Maetzler, W. (2022). Effect of lower limb vs. abdominal compression on mobility in orthostatic hypotension: A single-blinded, randomized, controlled, cross-over pilot study in Parkinson’s disease. Journal of Parkinson's Disease, 12(8), 2531-2541.		×			
71	Pfohl, M., Siegmund, T., Pscherer, S., Pegelow, K., & Seufert, J. (2015). Effectiveness and tolerability of treatment intensification to basal–bolus therapy in patients with type 2 diabetes on previous basal insulin-supported oral therapy with insulin glargine or supplementary insulin therapy with insulin glulisine: the PARTNER observational study. Vascular health and risk management, 569-578.		×			
72	Rabelo, H. T., Oliveira, R. J., & Bottaro, M. (2004). Effects of resistance training on activities of daily living in older women. Biol Sport, 21(4), 325-36.		×			

73	Ramos, J. S., Dalleck, L. C., Borrani, F., Beetham, K. S., Wallen, M. P., Mallard, A. R., ... & Coombes, J. S. (2017). Low-volume high-intensity interval training is sufficient to ameliorate the severity of metabolic syndrome. Metabolic syndrome and related disorders, 15(7), 319-328.	×				
74	Rebsamen, S., Knols, R. H., Pfister, P. B., & De Bruin, E. D. (2019). Exergame-driven high-intensity interval training in untrained community dwelling older adults: a formative one group quasi-experimental feasibility trial. Frontiers in physiology, 10, 449529.			×		×
75	Riaz, H., Babur, M. N., & Farooq, A. (2022). Effects of high-intensity multi-modal exercise training (HIT-MMEX) on bone mineral density and muscle performance in postmenopausal women. A Pilot randomized controlled trial.		×			
76	Rica, R. L., Shimojo, G. L., Gomes, M. C., Alonso, A. C., Pitta, R. M., Santa‐Rosa, F. A., ... & Bocalini, D. S. (2020). Effects of a Kinect‐based physical training program on body composition, functional fitness and depression in institutionalized older adults. Geriatrics & gerontology international, 20(3), 195-200.		×			
77	Richards, J. C., Johnson, T. K., Kuzma, J. N., Lonac, M. C., Schweder, M. M., Voyles, W. F., & Bell, C. (2010). Short‐term sprint interval training increases insulin sensitivity in healthy adults but does not affect the thermogenic response to β‐adrenergic stimulation. The Journal of physiology, 588(15), 2961-2972.	×				
78	Robinson, M. M., Lowe, V. J., & Nair, K. S. (2018). Increased brain glucose uptake after 12 weeks of aerobic high-intensity interval training in young and older adults. The Journal of Clinical Endocrinology & Metabolism, 103(1), 221-227.			×		×
79	Rodríguez, D. A., Arbillaga, A., Barberan‐Garcia, A., Ramirez‐Sarmiento, A., Torralba, Y., Vilaró, J., ... & Marco, E. (2016). Effects of interval and continuous exercise training on autonomic cardiac function in COPD patients. The clinical respiratory journal, 10(1), 83-89.					×
80	Rognmo, Ø., Moholdt, T., Bakken, H., Hole, T., Mølstad, P., Myhr, N. E., Grimsmo, J., & Wisløff, U. (2012). Cardiovascular risk of high- versus moderate-intensity aerobic exercise in coronary heart disease patients. Circulation, 126(12), 1436–1440.			×		×

81	Rohmansyah, N. A., Ka Praja, R., Phanpheng, Y., & Hiruntrakul, A. (2023). High-intensity interval training versus moderate-intensity continuous training for improving physical health in elderly women. INQUIRY: The Journal of Health Care Organization, Provision, and Financing, 60, 00469580231172870.	×				
82	Romain, A. J., Fankam, C., Karelis, A. D., Letendre, E., Mikolajczak, G., Stip, E., & Abdel-Baki, A. (2019). Effects of high intensity interval training among overweight individuals with psychotic disorders: a randomized controlled trial. Schizophrenia research, 210, 278-286.	×				
83	Rønnestad, B. R., & Hansen, J. (2016). Optimizing interval training at power output associated with peak oxygen uptake in well-trained cyclists. The Journal of Strength & Conditioning Research, 30(4), 999-1006.	×				
84	Sabag, A., Little, J. P., & Johnson, N. A. (2022). Low-volume high-intensity interval training for cardiometabolic health. The Journal of physiology, 600(5), 1013-1026.			×		×
85	Sackner, M. A., Lopez, J. R., Banderas, V., & Adams, J. A. (2020). Can physical activity while sedentary produce health benefits?			×		×
86	Sagarra-Romero, L., Vicente-Rodríguez, G., Pedrero-Chamizo, R., Vila-Maldonado, S., Gusi, N., Villa-Vicente, J. G., ... & Gómez-Cabello, A. (2019). Is sitting time related with physical fitness in Spanish elderly population? The EXERNET multicenter study. The Journal of nutrition, health and aging, 23(5), 401-407.			×		×
87	Saito, S., Washio, T., Watanabe, H., Ando, S., & Ogoh, S. (2021). Effect of intermittent isometric handgrip exercise protocol with short exercise duration on cognitive performance. The Journal of Physiological Sciences, 71(1), 12.			×		×
88	Sawashita, J., Onitsuka, S., Gen-no, H., Ishikawa, S., Iino, F., Tateishi, N., ... & Higuchi, K. (2009). Effects of mild calorie restriction and high-intensity interval walking in middle-aged and older overweight Japanese. Experimental gerontology, 44(10), 666-675.			×		×

89	Snijders, T., Nederveen, J. P., Bell, K. E., Lau, S. W., Mazara, N., Kumbhare, D. A., ... & Parise, G. (2019). Prolonged exercise training improves the acute type II muscle fibre satellite cell response in healthy older men. The Journal of physiology, 597(1), 105-119.			×		×
90	Søgaard, D., Baranowski, M., Larsen, S., Taulo Lund, M., Munk Scheuer, C., Vestergaard Abildskov, C., ... & Wulff Helge, J. (2019). Muscle-saturated bioactive lipids are increased with aging and influenced by high-intensity interval training. International Journal of Molecular Sciences, 20(5), 1240.			×		×
91	Søgaard, D., Lund, M. T., Scheuer, C. M., Dehlbaek, M. S., Dideriksen, S. G., Abildskov, C. V., ... & Helge, J. W. (2018). High‐intensity interval training improves insulin sensitivity in older individuals. Acta physiologica, 222(4), e13009.			×		×
92	Sosner, P., Gayda, M., Dupuy, O., Garzon, M., Lemasson, C., Gremeaux, V., ... & Bosquet, L. (2016). Ambulatory blood pressure reduction following high-intensity interval exercise performed in water or dryland condition. Journal of the American Society of Hypertension, 10(5), 420-428.			×		×
93	Støa, E. M., Meling, S., Nyhus, L. K., Strømstad, G., Mangerud, K. M., Helgerud, J., ... & Støren, Ø. (2017). High-intensity aerobic interval training improves aerobic fitness and HbA1c among persons diagnosed with type 2 diabetes. European journal of applied physiology, 117, 455-467.					×
94	Taylor, J. D., Fletcher, J. P., Mathis, R. A., & Cade, W. T. (2014). Effects of moderate-versus high-intensity exercise training on physical fitness and physical function in people with type 2 diabetes: a randomized clinical trial. Physical therapy, 94(12), 1720-1730.	×				
95	Thijssen, D. H., Benda, N. M., Kerstens, T. P., Seeger, J. P., Van Dijk, A. P., & Hopman, M. T. (2019). 12-week exercise training, independent of the type of exercise, attenuates endothelial ischaemia-reperfusion injury in heart failure patients. Frontiers in physiology, 10, 264.			×		×
96	Tsuda, K., Hayashi, E., Kamiya, K., Kudo, A., Kakihana, H., Nakayama, S., ... & Tamaki, J. (2022). Effects of interval-walking training on blood pressure in community-dwelling Japanese older adults. The Journal of Sports Medicine and Physical Fitness.			×		×

97	Uc, E. Y., Doerschug, K. C., Magnotta, V., Dawson, J. D., Thomsen, T. R., Kline, J. N., ... & Darling, W. G. (2014). Phase I/II randomized trial of aerobic exercise in Parkinson disease in a community setting. Neurology, 83(5), 413-425.			×		×
98	Villelabeitia-Jaureguizar, K., Vicente-Campos, D., Senen, A. B., Jiménez, V. H., Garrido-Lestache, M. E. B., & Chicharro, J. L. (2017). Effects of high-intensity interval versus continuous exercise training on post-exercise heart rate recovery in coronary heart-disease patients. International journal of cardiology, 244, 17-23.	×				
99	Vogel, T., Leprêtre, P. M., Brechat, P. H., Lonsdorfer, E., Benetos, A., Kaltenbach, G., & Lonsdorfer, J. (2011). Effects of a short-term personalized Intermittent Work Exercise Program (IWEP) on maximal cardio-respiratory function and endurance parameters among healthy young and older seniors. The Journal of nutrition, health and aging, 15(10), 905-911.			×		×
100	Warburton, D. E., McKenzie, D. C., Haykowsky, M. J., Taylor, A., Shoemaker, P., Ignaszewski, A. P., & Chan, S. Y. (2005). Effectiveness of high-intensity interval training for the rehabilitation of patients with coronary artery disease. The American journal of cardiology, 95(9), 1080-1084.	×				
101	West, M. A., Loughney, L., Lythgoe, D., Barben, C. P., Sripadam, R., Kemp, G. J., ... & Jack, S. (2015). Effect of prehabilitation on objectively measured physical fitness after neoadjuvant treatment in preoperative rectal cancer patients: a blinded interventional pilot study. British journal of anaesthesia, 114(2), 244-251.			×		×
102	Winding, K. M., Munch, G. W., Iepsen, U. W., Van Hall, G., Pedersen, B. K., & Mortensen, S. P. (2018). The effect on glycaemic control of low-volume high-intensity interval training versus endurance training in individuals with type 2 diabetes. Diabetes, Obesity and Metabolism, 20(5), 1131-1139.	×				
103	Wyckelsma, V. L., Levinger, I., McKenna, M. J., Formosa, L. E., Ryan, M. T., Petersen, A. C., ... & Murphy, R. M. (2017). Preservation of skeletal muscle mitochondrial content in older adults: relationship between mitochondria, fibre type and high-intensity exercise training. The Journal of physiology, 595(11), 3345-3359.			×		×
104	Yasar, Z., Elliott, B. T., Kyriakidou, Y., Nwokoma, C. T., Postlethwaite, R. D., Gaffney, C. J., ... & Hayes, L. D. (2021). Sprint interval training (SIT) reduces serum epidermal growth factor (EGF), but not other inflammatory cytokines in trained older men. European Journal of Applied Physiology, 121, 1909-1919.			×		×
